# Industrial Lignin Upcycled to High-Performance, Cost-Competitive Bio-based Adhesives via Green Ion-Exchange Self-Catalytic Strategy

**DOI:** 10.34133/research.1226

**Published:** 2026-04-09

**Authors:** Jiajun Liu, Hongcai Lu, Yuan Liu, Shi Liu, Wen Wang, Yongzhuang Liu, Qinqin Xia, Haipeng Yu

**Affiliations:** State Key Laboratory of Woody Oil Resources Utilization, Key Laboratory of Bio-based Material Science and Technology of Ministry of Education, Northeast Forestry University, Harbin 150040, China.

## Abstract

The global pulp industry discards over 50 million tons of lignin annually, with conventional valorization strategies hindered by performance and cost barriers. We report an ion-exchange, self-catalytic strategy to upcycle the industrial lignosulfonate into high-performance bio-based adhesives. The facile ion-exchange process transforms sodium lignosulfonate into lignosulfonic acid, which acts as not only a copolymer backbone of adhesive but also an intrinsic macromolecular catalyst, autonomously driving esterification reaction with citric acid and then wood polymers, eliminating the need of fossil-derived cross-linkers and energy-intensive pretreatments. This mechanism endows it performing excellent performance in bonding strength and boiling-water resistance. Crucially, the performance gains are coupled with important economic and environmental benefits; production costs are substantially lower than conventional petrochemical resins, and a life cycle assessment confirms markedly reduced environmental impacts alongside negligible formaldehyde emissions. This work successfully resolves the performance–sustainability–cost trade-off, establishing a waste-to-wealth pathway for circular manufacturing.

## Introduction

Traditional petrochemical adhesives such as urea–formaldehyde (UF) and phenol–formaldehyde (PF) resins offer superior performance but release hazardous formaldehyde and volatile organic compounds, causing over 3 million annual premature deaths globally and impeding sustainable manufacturing [1]. Bio-based adhesives derived from renewable resources (e.g., lignin, starch, and soy protein) present a promising alternative to reduce carbon footprints and fossil dependency [2–5].

Lignin is a natural “adhesive” in plant cell walls, forming a robust structural framework around microfibrils. Currently, less than 10% of the 50 million tons of lignin from pulp waste is valorized due to technological bottlenecks [6–8]. This underutilization has spurred growing interest in improving recovery processes and enabling high-value uses [9–12]. Lignin is particularly effective as a replacement for petrochemical-based phenolic compounds in adhesive production due to its aromatic structure [13–16]. Previous studies have demonstrated that chemical modifications of lignin, such as phenolation and epoxidation, can enhance the thermal stability and wet strength of lignin-based adhesives [17–20]. Despite significant advancements in lignin-based adhesives, several critical limitations remain: (a) dependence on fossil-derived cross-linkers (e.g., glutaraldehyde and epoxides), which undermines the “green” and “all-bio-based” claims [21,22]; (b) energy-intensive modification processes (e.g., depolymerization and functionalization) that increase costs and CO_2_ emissions [23]; and (c) emission of formaldehyde and volatile organic compounds [24]. As a result, truly high-performance, sustainable, and cost-competitive lignin-based adhesives have yet to be realized.

**Fig. 1.**
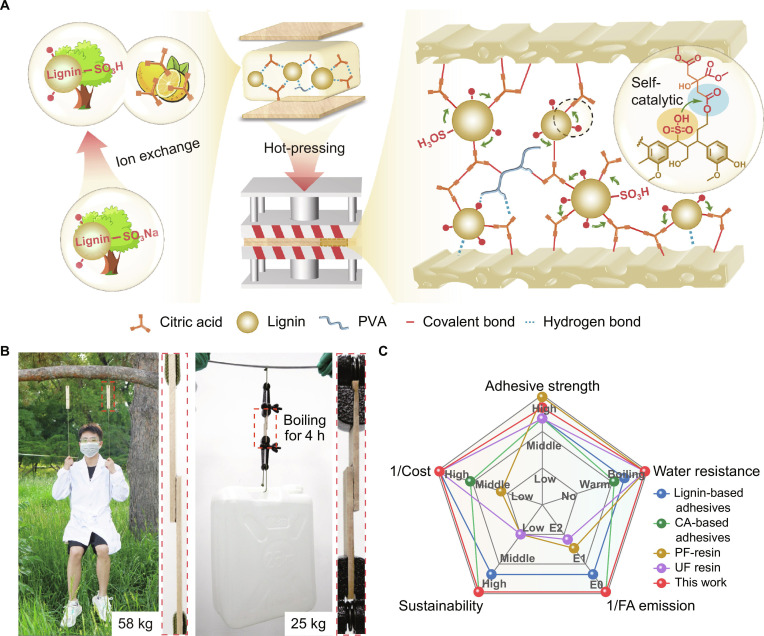
Schematic illustration of the lignosulfonic acid (LA)/citric acid (CA) adhesive’s preparation mechanism and performance. (A) Conversion of sodium lignosulfonate to LA via a facile, energy-efficient ion-exchange process. The in situ-generated LA acts as an intrinsic catalyst for esterification during hot pressing and curing, forming robust covalent bonds within the adhesive matrix and with the substrates. (B) Photograph of LA/CA-bonded wood panels supporting a 58-kg adult and a 25-kg bucket of water after 4 h of immersion in boiling water. (C) Radar chart comparing the LA/CA adhesive with previously reported lignin-based, CA-based, urea–formaldehyde (UF), and phenol–formaldehyde (PF) adhesives in terms of adhesive strength, water resistance, cost, sustainability, and formaldehyde emission.

To address the challenges, an ion-exchange, self-catalytic esterification strategy was proposed to convert industrial sodium lignosulfonate (SL) into active lignosulfonic acid (LA) (Fig. 1A). The core innovation lies in the integrative design of a self-catalytic cross-linking system where LA serves as both a copolymer backbone of adhesive and an intrinsic macromolecular catalyst [25,26]. LA would catalyze its covalent integration with other bio-based molecules without need of external fossil-based cross-linkers (e.g., glutaraldehyde and isocyanates). By combining LA with citric acid (CA), we formulated a fully bio-based adhesive that eliminates toxic catalysts and external energy inputs during synthesis. The LA/CA adhesive exhibits excellent bonding strengths especially the wet shear strength (Fig. 1B and Fig. S1). A comprehensive comparison with existing lignin-based, CA-based, and petrochemical adhesives (e.g., UF and PF resins) [25–36] highlights the LA/CA system’s advantages across sustainability, performance, cost competitiveness, and eco-friendliness (Fig. 1C and Fig. S2). This waste-to-adhesive paradigm offers a route to a circular bioeconomy, aligning with United Nations Sustainable Development Goals 9 (Industry Innovation and Infrastructure), 11 (Sustainable Cities and Communities), 12 (Responsible Consumption and Production), and 13 (Climate Action) through waste valorization and carbon footprint reduction.

## Results and Discussion

### Fabrication of the LA/CA adhesive

**Fig. 2.**
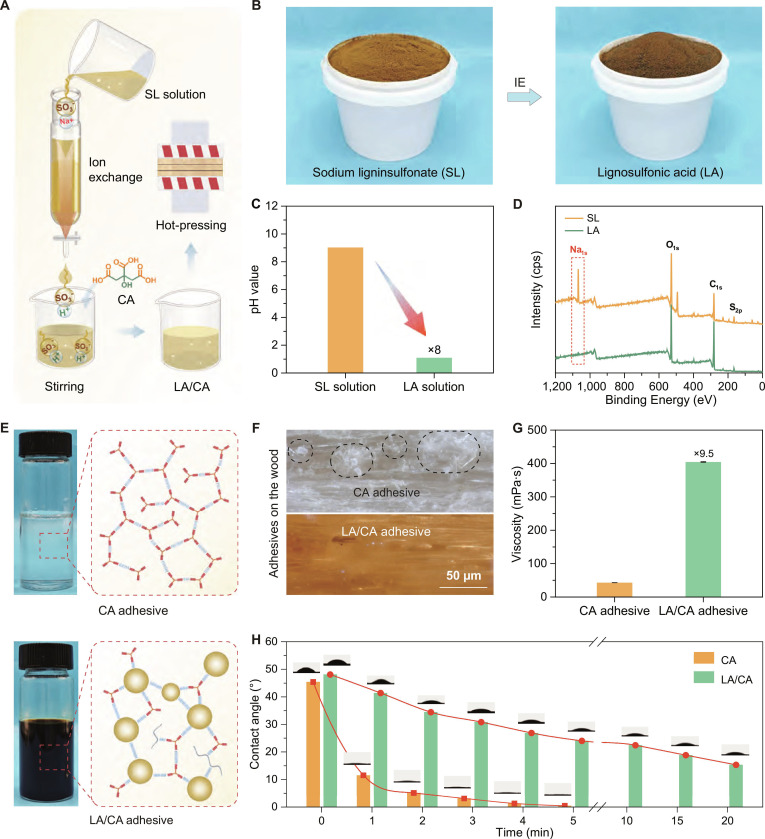
Fabrication process and property characterizations of the lignosulfonic acid (LA)/citric acid (CA) adhesive. (A) Schematic of the LA/CA adhesive fabrication process. (B) Photographs depicting the sodium lignosulfonate (SL) stage and the intermediate LA product. (C) Change of pH values from the SL to LA. (D) X-ray photoelectron spectroscopy (XPS) spectra analysis for both SL and LA. (E) Digital images and structural diagrams illustrating the CA and LA/CA adhesives. (F) Super depth-of-field microscope images of the CA and LA/CA adhesives applied to a wood surface. (G) Viscosity comparison between the CA and LA/CA adhesives. (H) Measurement of the contact angles of CA and LA/CA adhesives on a wood substrate over time.

The SL derived from pulp waste is low-cost and abundantly available. Similarly, CA is also renewable, nontoxic, and inexpensive. Accordingly, SL and CA were used as raw materials to prepare the cost-competitive bio-based adhesives (Fig. 2A). During the sulfite pulping process, lignin is sulfonated at the *C*_α_ position, primarily via cleavage of α-O-4’ or other ether bonds [37,38]. Since the sodium sulfonate group of SL lacks proton-donating capacity for acid-catalyzed esterification, SL was first dissolved in water (1:2 w/w) and then transformed into LA via a simple ion-exchange process (Fig. 2B and Fig. S3). The –ONa and –SO_3_Na groups were converted to –OH and –SO_3_H groups [39,40], causing the pH of the LA solution to drop from 9.03 to 1.10 (Fig. 2C and Fig. S4). X-ray photoelectron spectroscopy (XPS) spectra showed almost complete disappearance of the Na_1s_ peak at 1,067 eV, signaling the effective removal of sodium (Fig. 2D). The transformation of –SO_3_Na to –SO_3_H groups further confirmed by Fourier-transform infrared (FTIR) spectra and high-resolution S_2p_ spectra (Fig. S5A and B). Meanwhile, elemental analysis revealed that the ion exchange had minimal effect on the overall elemental compositions (Table S1). The above results indicate the successful preparation of LA.

^1^H–^13^C nuclear magnetic resonance (NMR) and gel permeation chromatography (GPC) spectra revealed no obvious changes in the structure and molecular weight between the SL and LA (Figs. S5C and S6). This indicated that the chemical structure of lignin remained intact during ion exchange. Differential scanning calorimetry (DSC) demonstrated that the glass transition temperature (*T*_g_) of LA is slightly higher than that of SL (Fig. S7A). Thermogravimetric analysis (TGA) and derivative thermogravimetric (DTG) curves (Fig. S7B and C) displayed that LA displayed slightly lower thermal stability than SL, but no significant changes. These comprehensive analyses illustrate that ion exchange primarily involved the conversion of ionic groups without disrupting the main structure and properties of lignin molecules [41]. The remaining aromatic structure of lignin would be beneficial for enhancing the thermal stability of adhesives.

CA’s environmental friendliness makes it an attractive wood adhesive component. However, when used alone, CA penetrates wood substrates quickly, preventing the formation of a continuous adhesive film. This excessive penetration wastes adhesive, compromising bond strength and water resistance. To address this issue, we blended LA with CA to form a synergistic hydrogen bond network (Fig. 2E). The resulting LA/CA exhibited increased surface tension and viscosity. Unlike the CA-only formulation, which failed to form a continuous glue layer on the wood surface (Fig. 2F), the LA/CA adhesive could achieve continuous glue layer (Figs. S8 and S9). Viscosity measurements (Fig. 2G) showed that the LA/CA is 9.5 times more viscous than neat CA. Varying the solid content of CA adhesive from 55 to 70 wt % only modestly increased viscosity (from 17.1 to 68.6 mPa·s; Fig. S10 and Table S2). In contrast, the viscosity of LA/CA rose dramatically from 62.4 to 8,393 mPa·s with increasing solid content (Table S3). The high molecular weight and 3-dimensional aromatic polymer network formed by LA and CA underlie this substantial viscosity enhancement. The positive correlation between LA content and viscosity confirms the crucial role of LA in constructing the intermolecular network.

Time-resolved contact angle measurements on wood substrate (Fig. 2H) further elucidate wetting and penetration behaviors. The CA adhesive displayed an initial contact angle of 45°, which rapidly declined, indicating fast spreading and deep substrate infiltration. By contrast, the LA/CA adhesive started at 48° and remained relatively stable over time, demonstrating controlled wetting without excessive penetration. Furthermore, the LA/CA adhesive demonstrated good storage stability at 4 to 25 °C and shelf life. It still maintained excellent bonding strength even after being stored for 120 d (Fig. S11).

### Thermal stability and bonding property of the LA/CA adhesive

**Fig. 3.**
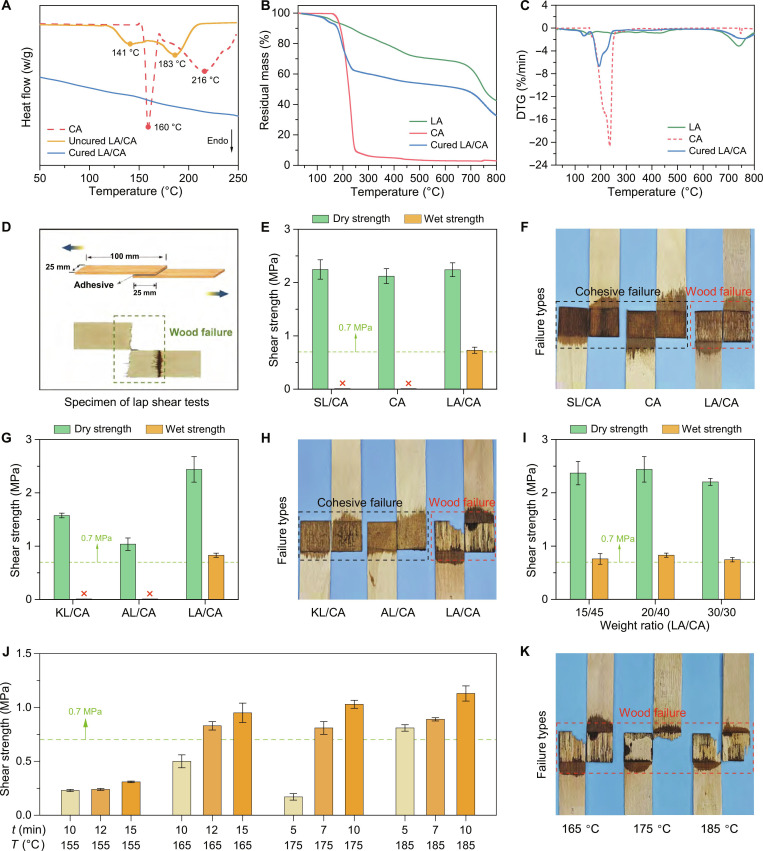
Thermal stability and adhesive performance of the lignosulfonic acid (LA)/citric acid (CA) system. (A) Differential scanning calorimetry (DSC) thermograms of CA, uncured LA/CA blend, and cured LA/CA. (B) Thermogravimetric analysis (TGA) and (C) derivative thermogravimetric (DTG) curves of LA, CA, and cured LA/CA. (D) Schematic of the lap-shear test specimen and the wood-failure appearance after the dry shear strength test. (E) Comparison of dry and wet shear strengths for sodium lignosulfonate (SL)/CA, CA, and LA/CA adhesives. Dashed line denotes the Type I minimum requirement (GB/T 17657-2022). (F) Representative failure modes following wet shear tests of SL/CA, CA, and LA/CA. (G) Dry and wet shear strengths for KL/CA, AL/CA, and LA/CA (Type I threshold shown). (H) Representative failure modes after wet shear tests of KL/CA, AL/CA, and LA/CA. (I) Dry and wet shear strengths of adhesives with varying LA/CA ratios. (J) Wet shear strength of LA/CA-bonded specimens as a function of hot-pressing temperature and time. (K) Failure modes of specimens pressed at 165, 175, and 185 °C after wet shear testing.

Hot pressing and curing are essential to improve the mechanical strength and water resistance of adhesives. To elucidate the thermal behavior of CA, uncured LA/CA, and fully cured LA/CA, we performed DSC analysis (Fig. 3A). The DSC trace for neat CA showed an endothermic event at 160 °C, corresponding to melting and dehydration to form anhydride, followed by thermal decomposition at 216 °C. In contrast, the LA/CA adhesive exhibited 2 shifted endothermic peaks at 141 and 183 °C. The 141 °C transition marked the onset of covalent cross-linking, while the 183 °C peak reflected further esterification of CA. After curing, the LA/CA sample displayed no discernible endothermic or exothermic peaks, indicating near-complete reaction. TGA and DTG (Fig. 3B and C) confirmed that CA alone has limited thermal stability, whereas the inclusion of LA markedly enhances the thermal robustness.

We assessed bonding strength and water resistance via lap-shear tests on specimens hot-pressed at 165 °C for 12 min (Fig. 3D). Both SL/CA and CA adhesives achieved the GB/T 17657–2022 dry shear strength threshold (0.7 MPa) but failed under boiling condition (Fig. 3E and Fig. S12). Raising the hot-pressing temperature to 180 to 200 °C did not remedy this deficiency (Fig. S13). By contrast, the LA/CA adhesive satisfied both dry and wet strength criteria, with further gains realized through optimized hot-pressing parameters (Fig. 3F and Fig. S14). Addition of polyvinyl alcohol (PVA) could increase wet shear strength, likely because the flexible chains of PVA enhanced the cohesion of the adhesive (Fig. S15). Furthermore, the formula was optimized by using PVA with a molecular weight of 1,788 and the quantity of 0.5 wt % (Fig. S16).

To explore the generality of lignin sources, we prepared kraft lignin/citric acid (KL/CA) and alkali lignin/citric acid (AL/CA) adhesives. Although both formulations met the minimum dry shear requirement (Fig. 3G), they suffered cohesive failure and did not withstand wet testing (Fig. 3H and Fig. S17). FTIR and XPS spectra analyses indicated that KL/AL contains almost no –SO_3_H groups, thus failing to provide the proton catalytic sites required for the esterification reaction, resulting in poor bonding performance (Fig. S18). This further emphasizes the superior catalytic effect of the LA sulfonic acid group in promoting esterification upon the hot pressing.

We also prepared sulfonated kraft lignin and sulfonated alkali lignin and prepared adhesives with them (Fig. S19). The results showed that the bonding strengths of the adhesives prepared from sulfonated kraft lignin and sulfonated kraft lignin were similar to that of LA/CA adhesive, and the wet shear strength also met the Type I standard. This indicates that the self-catalytic esterification strategy in this study is not only applicable to lignosulfonate raw materials but also that other industrial lignin can achieve the same application effect through simple sulfonation modification.

We further investigated the role of sulfonic acid by doping *p*-toluenesulfonic acid (TsOH) to CA and SL/CA adhesives, at a similar –SO_3_H content (~1.36 mmol/g). Although CA-TsOH (4 to 5 wt %) and SL/CA-TsOH (4 to 5 wt %) met the Type I standard, their overall performance lagged the LA/CA adhesive (Fig. S20). The cross-linking density and bonding strength are positively correlated with the –SO_3_H density (Fig. S21 and Table S4). However, the incorporation of TsOH would raise the adhesive production cost by 70.80 to 88.50 €/ton, whereas the LA/CA adhesive holds economic advantages.

By varying the LA/CA ratio and hot-pressing conditions, we identified an optimal mass ratio of 20/40, yielding peak dry shear strengths of 2.20 to 2.44 MPa and wet shear strengths of 0.75 to 0.83 MPa (Fig. 3I). Hot pressing at 155 °C, even with extended dwell times, failed to achieve the 0.70 MPa wet strength threshold, indicating incomplete curing (Fig. 3J). Failure-mode analysis corroborated these temperature-dependent effects (Fig. 3K). At 165 °C for ≥12 min, both shear strengths improved (Fig. S22). Further raising the temperature to 175 °C (≥7 min) or 185 °C (≥5 min) produced wet shear strengths of 0.81 to 1.13 MPa and resulted in wood failure (Fig. 3K). These findings demonstrate that a hot-pressing temperature of 165 °C is required for full curing and that further increases in temperature or time accelerate esterification and reinforce adhesive cross-linking. The LA/CA adhesive not only can be used for bonding poplar wood but also maintains excellent performance when bonding other types of wood (e.g., pine wood and birch wood), indicating good applicability of the wood (Fig. S23A). Moreover, the incorporation of suitable curing agents can lower the industrial pressing temperature to 150 °C without compromising performance, thereby compatible with existing production lines (Fig. S23B).

Finally, we compared the wet shear strength of this LA/CA adhesive with other bio-based adhesives reported in recent studies [25–36] (Fig. S24 and Table S5). The LA/CA formulation not only fulfills but also significantly exceeds Type I standards, achieving a wet strength ~1.6 times the requirement and 100% wood failure. Such boiling-water resistance is uncommon among bio-based adhesives, underscoring the LA/CA competitiveness of adhesive among state-of-the-art systems.

### Bonding mechanism of the LA/CA adhesive

**Fig. 4.**
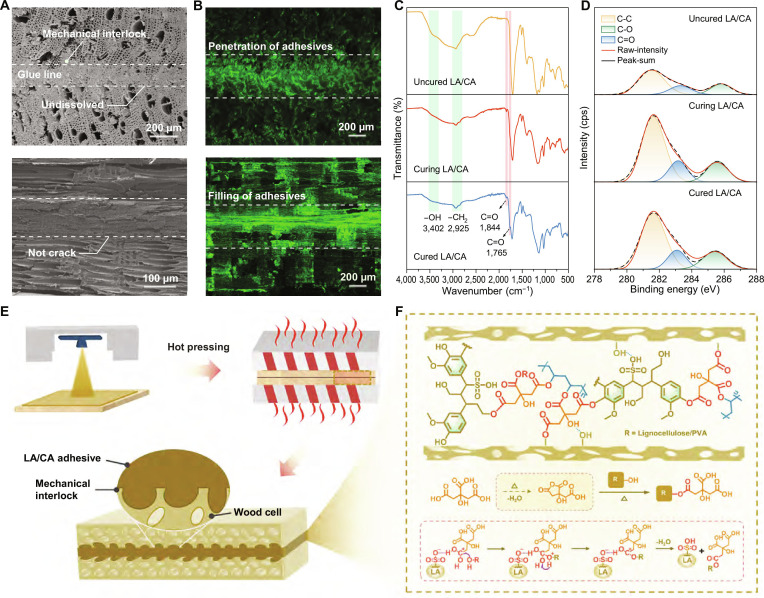
Bonding mechanism of the lignosulfonic acid (LA)/citric acid (CA) adhesive. (A) Scanning electron microscopy (SEM) images of cross-sectional and longitudinal plywood specimens after boiling water treatment. (B) Fluorescence confocal microscopy images of the corresponding sections. (C) Fourier-transform infrared (FTIR) spectra of LA/CA in the uncured, curing, and fully cured states. (D) X-ray photoelectron spectroscopy (XPS) spectra of LA/CA at each curing stage. (E) Schematic diagram of adhesive–wood veneer interfacial interactions. (F) Proposed chemical bonding mechanism between LA/CA adhesive and lignocellulosic hydroxyl groups during wood bonding.

The bonding strength of wood adhesives is governed by both physical and chemical interactions between the adhesive and the wood substrate [42]. Physically, the adhesive penetrates and fills the vessel pores of the veneer, forming mechanical interlocks. Figure 4A shows that the LA/CA adhesive creates a continuous layer within the pores; after immersion in boiling water, no interfacial separation is observed, and the layer remains intact (Fig. S25). Confocal laser scanning microscopy images (Fig. 4B) further confirm uniform adhesive distribution and a robust veneer–adhesive interface.

Chemically, covalent interactions enhance bonding performance and water resistance. FTIR spectra reveal that during the curing process, the C=O absorption peak of CA at 1,708 cm^−1^ diminishes while a new peak appears at 1,844 cm^−1^, corresponding to cyclic anhydride formation via partial dehydration of CA [43,44]. In the fully cured LA/CA system, peaks associated with hydroxyl groups and anhydride C=O decrease and a distinct ester-bond peak emerges at 1,765 cm^−1^, indicating covalent cross-linking between LA and CA (Fig. 4C). XPS supports this conclusion by showing increased C=O and C–C signals in the cured LA/CA adhesive (Fig. 4D) [44,45]. By contrast, the cured SL/CA adhesive exhibits no new ester-bond peaks (Fig. S26) and poor water resistance, reflecting insufficient covalent bonding during hot pressing. Furthermore, the LA/CA achieved a conversion rate of nearly 96% at 12 min (reaching 75% within just 4 min). In contrast, the neat CA system reached only 20% conversion at 12 min and less than 5% at 4 min (Fig. S27). This 5-fold increase in reaction rate provides direct evidence for the self-catalytic esterification strategy enabled by LA.

Surface wettability is crucial for maximizing contact, diffusion, and adsorption at the adhesive–wood interface. The wetting property of LA/CA adhesive stems from its abundant polar carboxyl and hydroxyl groups, which interact well with natural wood (Fig. 4E). We propose 2 esterification pathways during curing: (a) CA dehydration to citric anhydride [46,47] and (b) direct esterification of CA with wood hydroxyl groups (Fig. 4F). In both pathways, LA acts as a catalyst: Its sulfonic acid groups protonate the carbonyl oxygen of CA or its anhydride, increasing the electrophilicity of the carbonyl carbon. Subsequent nucleophilic attack by an alcohol oxygen yields a tetrahedral intermediate, followed by intramolecular proton transfer and dehydration to regenerate the catalyst and form the ester bond (Fig. S28) [48]. In contrast, the sodium sulfonate groups in SL lack proton-donating ability and thus cannot catalyze these esterification reactions. Consequently, the functional groups in LA/CA not only enhance surface wettability and viscosity but also promote covalent cross-linking, resulting in enhanced bonding strength and boiling-water resistance.

### Scalability and environmental impacts

**Fig. 5.**
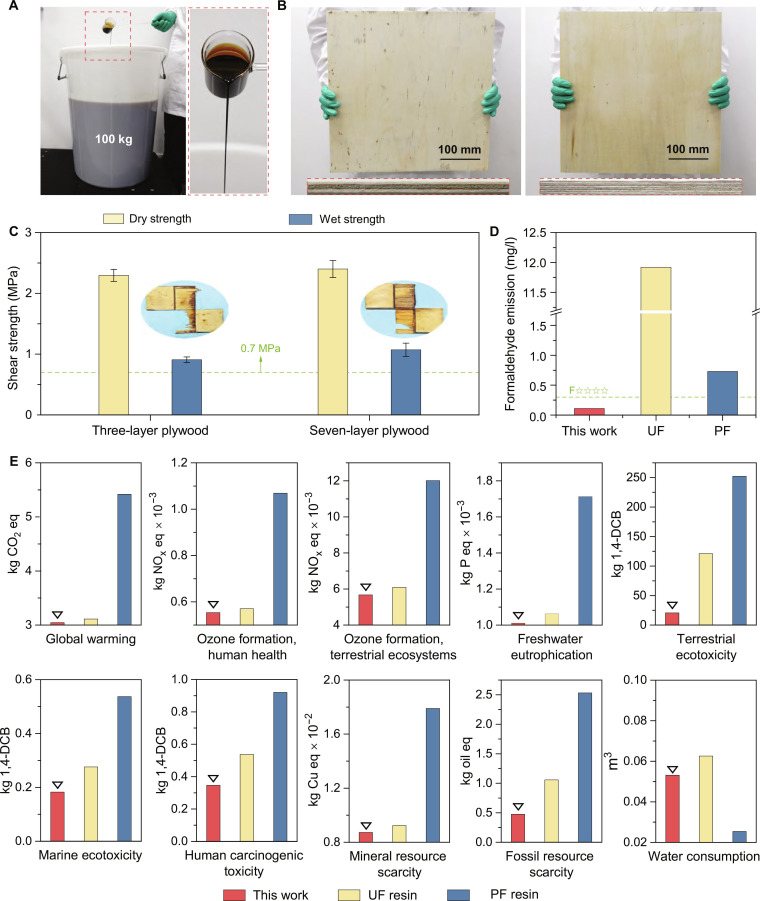
Scale-up production and life cycle assessment (LCA) analyses of the lignosulfonic acid (LA)/citric acid (CA) adhesive. (A) Photographs of the 100-kg pilot-scale production of LA/CA adhesive. (B) Photographs of 3-layer and 7-layer plywood panels bonded with the scaled-up adhesive. (C) Dry and wet shear strengths of the 3-layer and 7-layer panels. (D) Formaldehyde emissions from LA/CA versus urea–formaldehyde (UF) and phenol–formaldehyde (PF) adhesives. (E) Comparative life cycle environmental impacts of LA/CA, UF, and PF adhesives. Toxicity potentials are expressed in 1,4-dichlorobenzene equivalents, with which as the reference substance for toxicity characterization.

The streamlined production process of the LA/CA adhesive proves its suitability for industrial scale-up. We produced 100 kg of adhesive using industrial-grade SL and CA (Fig. 5A). Three- and 7-layer plywood panels bonded with this batch met the Type I standard (GB/T 17657–2022) for both dry and wet shear strength (Fig. 5B and C). The economic and sensitivity analyses revealed that the cost of LA/CA adhesive is approximately €279.32 per ton (Fig. S29 and Tables S6 to S8). A comparison of the costs for producing 1 m^3^ of plywood using this adhesive versus common commercial adhesives (UF, PF, and methylenediphenyl diisocyanate [MDI]) indicated that the LA/CA adhesive offers significant cost reductions of 19.7%, 46.2%, and 69.6% relative to UF, PF, and MDI, respectively (Fig. S30 and Table S9). Further optimization of the formulation is anticipated to improve both cost-effectiveness and competitiveness.

The corrosivity of the adhesive and its long-term acidity effects were also evaluated. Steel corrosion tests showed that the LA/CA adhesive caused no detectable corrosion (Fig. S31). This resistance is due to the nonoxidizing character of the organic sulfonic acid and the corrosion-inhibiting properties of lignosulfonate, which adsorbs onto metal surfaces to form a protective layer [49–51]. Upon curing, esterification consumes free protons and raises the adhesive-layer pH to 4.74, substantially reducing the risk of acid-mediated wood fiber degradation and ensuring long-term bond durability.

Environmental assessments further underscore the eco-friendliness of the LA/CA adhesive. Formaldehyde emissions were negligible (0.11 mg·l^−1^), meeting Japan’s ultra-low F^****^ classification (Fig. 5D). A life cycle assessment (LCA) conducted according to ISO 14040/14044 [52,53] compared the LA/CA with PF and UF resins (Fig. 5E and Fig. S32). The LA/CA exhibited lower impacts across all categories than UF and substantially lower impacts than PF, except in water consumption. Upstream raw-material production accounted for most of the environmental impact of LA/CA, while the actual adhesive preparation process only accounts for a small portion. This is mainly due to the SL derived from pulp waste, which has a significant environmental impact. However, our utilization of this waste lignin actually reduces the environmental pollution. The uncertainty analysis confirmed the reliability of the results (Table S10). These findings highlight the environmental sustainability and commercial potential of this LA/CA adhesive.

## Conclusion

This study introduced a straightforward and economical way to valorize industrial lignosulfonate into reactant macromonomers, paving the way for a high-performance, boiling-water-resistant bio-based adhesive. The core innovation of this work lies in the integrative design of a self-catalytic adhesive system where LA serves as an intrinsic macromolecular catalyst and copolymer. This self-catalytic mechanism is crucial, as it eliminates the need for external, often toxic catalysts such as strong mineral acids or glutaraldehyde. Additionally, LA serves as an intrinsic catalyst, promoting in situ esterification between the adhesive components CA and wood polymers during hot-pressing and curing process. LA synergizes with CA through covalent bond cross-linking, precisely controlling the adhesive’s viscosity to prevent excessive penetration and ensure the formation of a continuous glue layer. The covalent cross-links formed as a result contribute to the adhesive’s high wet strength, enabling the LA/CA adhesive to exceed the Type I standard, a benchmark that many bio-based adhesives have struggled to achieve.

Crucially, our approach addresses the long-standing trade-offs among performance, cost, and sustainability. This LA/CA adhesive is derived entirely from renewable biomass, effectively eliminating formaldehyde emissions. Furthermore, the process circumvents energy-intensive pretreatment or chemical modification of lignin (e.g., depolymerization and phenolation), thereby drastically reducing the embodied energy and carbon footprint. The LCA unequivocally confirms this advantage, showing a reduction of 28% to 63% in environmental impacts across most categories compared to petroleum-based resins. The economic analysis is equally compelling, demonstrating a 19% to 69% reduction in production costs compared to UF, PF, and MDI adhesives. This cost-effectiveness, primarily attributed to the use of waste-derived lignin and the catalyst-free, room-temperature preparation process, provides a tangible economic incentive for industry adoption. When contextualized within the broader field of bio-based adhesives, our strategy stands out for its simplicity and scalability.

In conclusion, this work establishes a waste-to-wealth paradigm that aligns with the principles of circular bioeconomy. It offers a scalable and commercially viable solution that reduces reliance on fossil resources, mitigates environmental pollution, and adds value to the forest product industry. Future work will focus on further optimizing the formulation for different wood composite applications. We believe this green and practical strategy represents a forward step toward sustainable manufacturing and holds promise for the large-scale utilization of industrial lignin.

## Methods

### Materials

SL (≥96.0%), CA (≥99.5%), and ion-exchange resin Amberlite 732 were purchased from Aladdin Reagent (Shanghai, China). Polyvinyl alcohol 1788 (PVA) (alcoholysis degree: 87.0% to 89.0%) was purchased from Zhonglian Reagent (Tianjin, China). All the above-mentioned chemicals were used without further purification.

### Ion-exchange procedure

Initially, the SL solution was prepared by dissolving 21 g of SL in 42 g of distilled water. The SL solution was then passed through the column containing Amberlite 732 cation-exchange resin at 10 ml/min flow rate at room temperature, thereby obtaining the ion-exchanged LA solution (~60 g). The pH of the solution decreased to 2.0, indicating that the cation was successfully exchanged. The capacity of the ion-exchange resin is 4.28 mmol/g, and it can be reused 50 to 70 times.

### Preparation of the LA/CA adhesive

The 40.0 g of CA (and 0.5 g of PVA) was gradually added to the LA solution under stirring to obtain the LA/CA adhesive with a solid content of 60 wt %. The fabrication process for adhesives with varying LA-to-CA ratios (15/45, 20/40, and 30/30) was carried out in the same manner as described above.

### Fabrication process of the SL/CA adhesive

SL (20 g) and 40 g of CA were gradually added to 40 g of distilled water. The mixture was then blended to produce the SL/CA adhesive, with a solid content of 60 wt %..

### Characterization and tests

#### Mechanical property tests

Poplar veneers with the size of 100 × 25 × 2 mm were loaded with the LA/CA adhesive over an area of 25 × 25 mm^2^ using 160 g·m^−2^ loading concentrations. Then, 2 pieces of poplar veneers were put into contact with one another. The specimen was hot-pressed at 155 to 185 °C for 5 to 15 min at 2 MPa to prepare the samples for shear strength test.

The specimens were pretreated in accordance with the Type I plywood standard specified in GB/T 17657–2022 to evaluate wet shear strength of the adhesive. The lap specimens were first soaked in boiling water for 4 h and then dried in an air-circulating oven at 63 °C for 20 h. Subsequently, the lap specimens were soaked in boiling water again for 4 h, followed by soaking in water at room temperature for 1 h. The pretreated lap specimens were then subjected to lap shear strength testing. All lap shear strength tests were conducted using a universal testing machine (SUNS, UTM2503). Three identically prepared samples were measured and averaged to ensure accuracy.

Three poplar veneers (100 × 25 × 2 mm) were used to fabricate a 3-layer plywood, with each layer oriented perpendicular to the grain direction of the adjoining layers. The LA/CA adhesive with a loading of approximately 160 g·m^−2^, was uniformly applied to both sides of the middle veneer. The veneers were then assembled and hot-pressed at 175 °C under 2 MPa for 7 min to prepare the 3-layer plywood. The preparation of the 7-layer plywood follows the same procedure as that of the 3-layer plywood. The shear strength of the plywood was tested in accordance with GB/T 17657–2022. The wet strength testing was conducted using the same procedure as described above for the lap shear specimens. Three identically prepared samples were measured and averaged.

#### FTIR spectroscopy

The FTIR spectroscopy with attenuated total reflectance module was used to characterize the LA/CA adhesive and lignin. The measurements were done right after sample preparation using a Spectrum Two FT-IR (Thermo Scientific Nicolet iS50 FTIR spectrometer), and the background extraction was done in air. The sample was dried before testing. The resolution was 1 cm^−1^, and 10 scans were collected for each measurement between 4,000 and 600 cm^−1^.

#### XPS analysis

An XPS system (Thermo Scientific K-Alpha, USA) was used to determine the chemical compositions of SL, LA, and LA/CA samples at surfaces. XPS survey scans at 80- and 1-eV steps were used to investigate the Na_1s_, O_1s_, C_1s_, and S_2p_ regions, whereas high-resolution scans at 50- and 0.1-eV steps were utilized to identify specific chemical bonding of the samples using a deconvolution approach with a Gaussian function.

#### ^1^H–^13^C NMR spectroscopy

The lignin sample (30 mg) was dissolved in 600 μl of D_2_O. The 2-dimensional Heteronuclear Single Quantum Coherence (HSQC)-NMR spectra were acquired from the same samples with a Bruker standard pulse sequence. The spectral widths were 5,000 and 20,000 Hz for the ^1^H and ^13^C dimensions. A total of 1,024 collected points were used for the ^1^H dimension with a recycle delay of 1.5 s, while 64 and 256 transients were used for the ^13^C dimension.

#### GPC analysis

Dried lignin samples were dissolved in distilled water (2 mg/ml), filtered through 0.22-μm polytetrafluoroethylene filters, and analyzed by high-performance liquid chromatography. The GPC system consisted of an Agilent PL-GPC 50 system. Eluting solvent was distilled water (1 ml/min), and column eluent was monitored by an Agilent 1260 diode array detector, at 280 ± 4 nm. The columns used were PLgel 5 μm Guard (50 × 7.5 mm), PLgel 5 μm MIXED-C (300 × 7.5 mm), and PLgel 3 μm MIXED-E (300 × 7.5 mm) in tandem, and the injection volume was 20 μl. The weight-average molecular weight (*M*_w_) value of lignin was calculated using the polystyrene standard sample with average *M*_w_ of 640, 1,230, 1,880, 4,720, 6,580, 9,580, 22,130, and 27,500 (g/mol) as standard curves. *M*_w_ was calculated using Agilent GPC software, the GPC curve was split by Peakfit software, and the log-normal amp was used for fitting to obtain the experimental results.

#### TGA

TGA and DSC analysis was performed on a NETZSCH STA 449 F5 thermogravimetric analyzer using a heating rate of 10 °C/min from 20 to 800 °C, with balance and sample purge rates set to 20 ml/min or nitrogen.

#### SEM analysis

The plywood specimens were cut into small pieces (4 × 4 × 4 mm) using a blade to prepare the test samples. A Phenom scanning electron microscopy (SEM) (JSM-7500F) with a resolution of 30 nm was used to analyze the surface morphology of the glue line region of the samples. The samples were coated with gold via electrodeposition prior to SEM analysis.

#### Viscosity test

The viscosity of the adhesive was obtained by rotational viscosimeter (NDJ-1B, Shanghai Changji). Each adhesive was tested 3 times and averaged.

#### Contact angle test

The contact angle was tested by a contact angle meter (Attension Theta, Biolin Scientific, Sweden). The adhesives were deposited on the surface of the wood, and the photograph of the drop image was recorded.

#### Formaldehyde emission test

The formaldehyde emission of plywood was tested according to the Chinese National Standard (GB/T 17657–2022) using the desiccator method.

#### Measurement of –SO_3_H group content

First, 0.10 g of LA was taken and dissolved in 20 ml of an aqueous sodium chloride solution at a concentration of 0.05 mol/l. The mixture was stirred and allowed to dissolve thoroughly at room temperature for 30 min. Subsequently, the solution was titrated with a 0.1 mol/l aqueous sodium hydroxide solution, using phenolphthalein as the indicator.

$$H=\left(V_{\text{NaOH}}\times C_{\text{NaOH}}\right)/m_{\mathrm{LA}}$$ (1)

#### LCA

This study systematically compared the environmental impact of LA/CA adhesive and UF and PF resin through LCA, following the ISO 14040/14044 standard framework. The system boundary is set from cradle to gate, covering upstream processes such as raw material acquisition, chemical production, energy supply, and resin synthesis (Fig. S32). The allocation issue is handled using the cutoff method. The modeling data come from laboratory-scale pilot processes, including materials, energy, and chemical input (Table S11). The background data mainly come from the internationally recognized Ecoinvent database, ensuring the reliability and comparability of the inventory data. The modeling platform selects the internationally mainstream LCA software Simapro. The life cycle impact assessment adopts the ReCiPe 2016 Midpoint (H) V1.10/World (2010) H method package, selecting 10 indicators, including global warming potential, ozone formation, human health, ozone formation, terrestrial ecosystems, freshwater eutrophication, marine eutrophication, terrestrial ecotoxicity, human carcinogenic toxicity, mineral resource scarcity, fossil resource scarcity, and water consumption. The calculation process covers characterization and normalization steps.

## Data Availability

The data that support the findings of this study are available within the article and the Supplementary Materials file. All other relevant source data are available from the corresponding authors upon request.
