# Supplementary material for: Industrial Lignin Upcycled to High-Performance, Cost-Competitive Bio-based Adhesives via Green Ion-Exchange Self-Catalytic Strategy
Source: Research (Wash D C). 2026 Apr 9;9:1226. doi: 10.34133/research.1226 (PMC13062486; doi:10.34133/research.1226)
Supplement: Supplementary 1 — Figs. S1 to S32 Tables S1 to S11 [file research.1226.f1.pdf]

Supplementary Information

Industrial Lignin Upcycled to High-Performance, Cost-Competitive Biobased Adhesives via Green Ion-Exchange Self-Catalytic Strategy

Jiajun Liu[1], Hongcai Lu[1], Yuan Liu[1], Shi Liu[1], Wen Wang[1], Yongzhuang Liu[1]*, Qinqin Xia[1]* and Haipeng Yu[1]*

1. State Key Laboratory of Woody Oil Resources Utilization; Key Laboratory of Bio-based Material Science and Technology of Ministry of Education, Northeast Forestry University, Harbin 150040, China.

*Email: *yuhaipeng20000@nefu.edu.cn*; *2018xiaqinqin@nefu.edu.cn*; *lyz@nefu.edu.cn*

Supplementary Figures: Fig. S1–S32

Supplementary Tables: Table S1–S11

1. Supplementary Figures

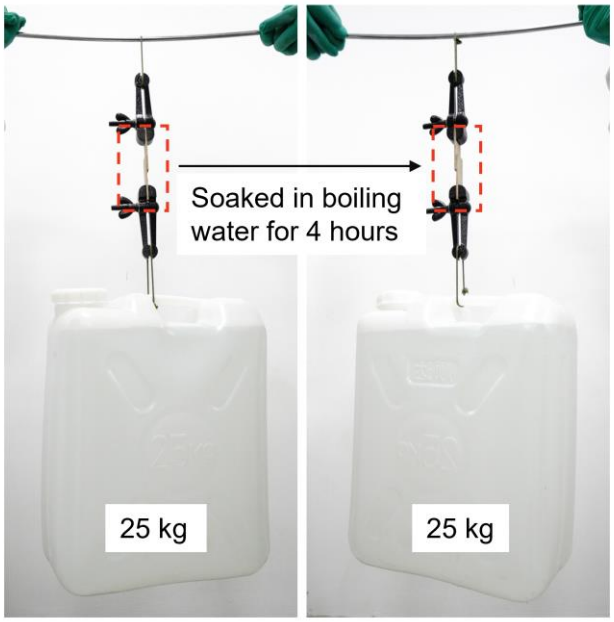

Fig. S1. Image of the LA/CA adhesive bonded wood sheets to support a bucket of water weighs 25 kg after soaking in boiling water for 4h with a bonding area of 25 $\times$ 25 mm^2.

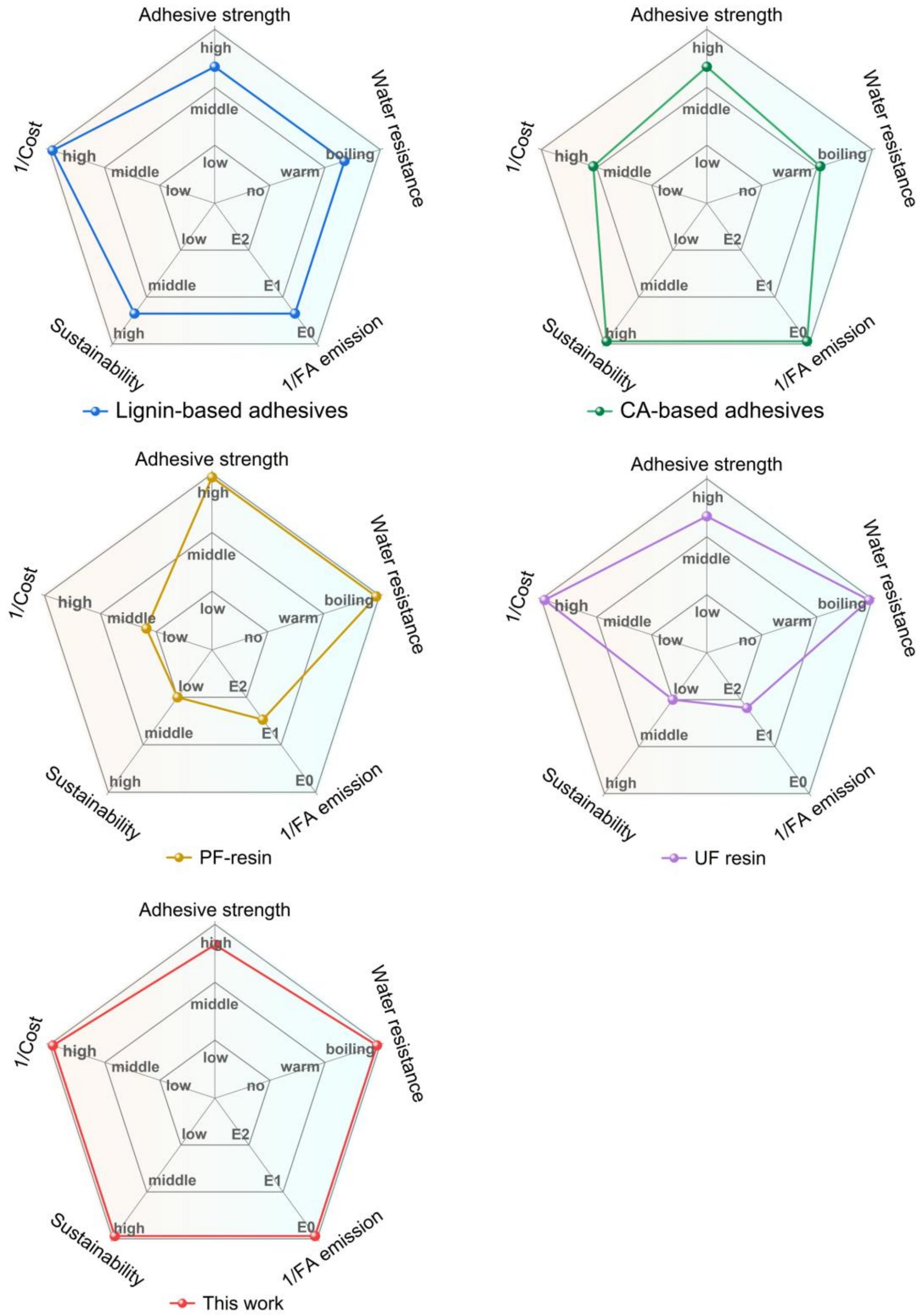

Fig. S2. Rough comparison between this work and previously reported most of lignin-based, citric acid-based adhesives, UF resin and PF resin in terms of bonding strength, cost, water resistance, sustainability and formaldehyde (FA) emissions.

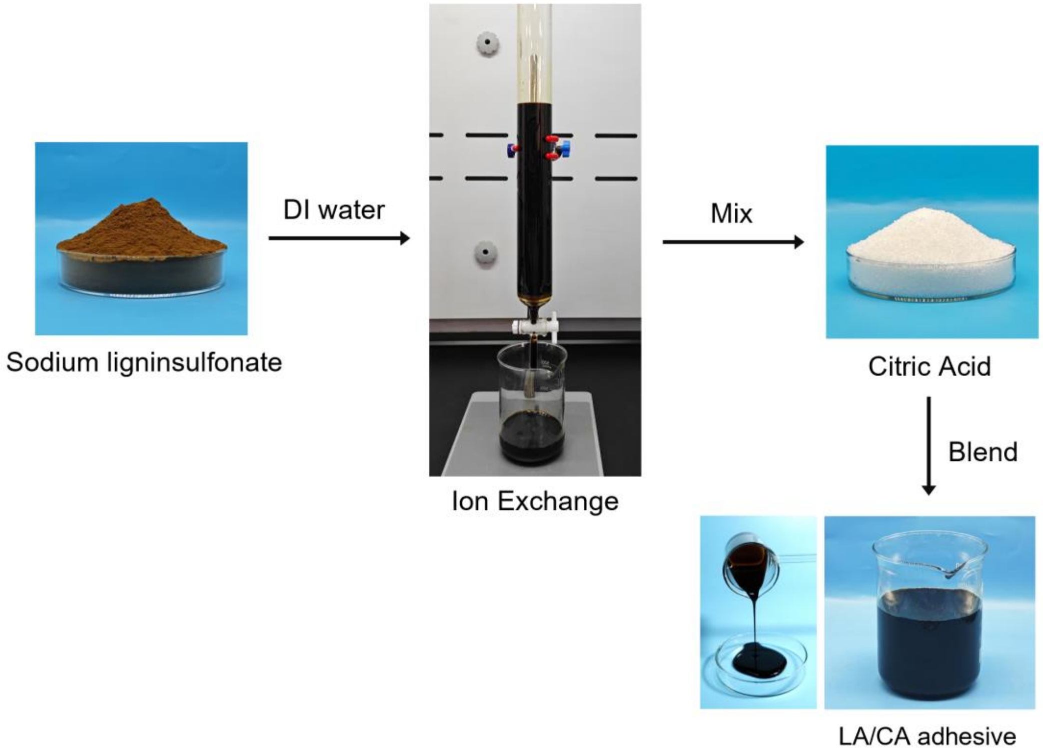

Fig. S3. The fabrication process of the LA/CA adhesive used in this work.

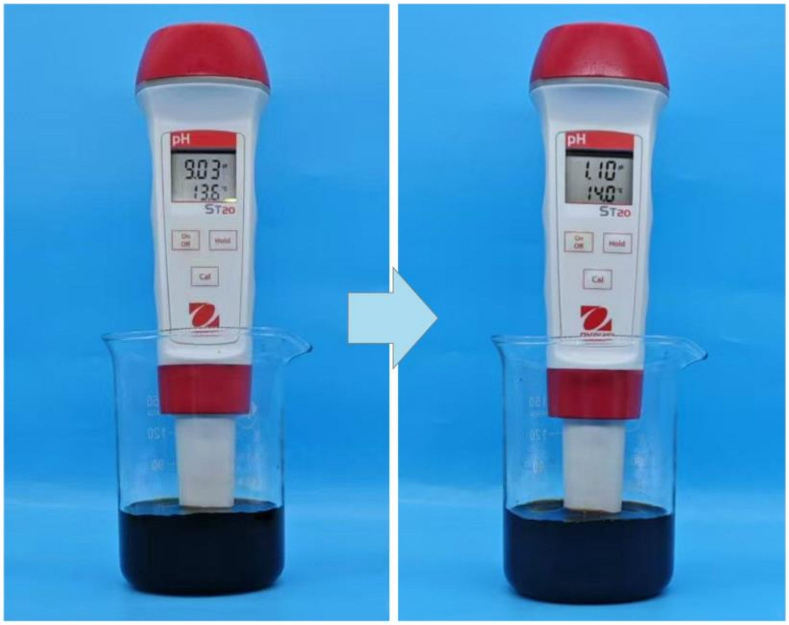

Sodium lignosulfonate (SL) Lignosulfonic acid (LA)

Fig. S4. The pH values of the sodium lignosulfonate (SL) solution and the ion-exchanged lignosulfonate (LA) solution.

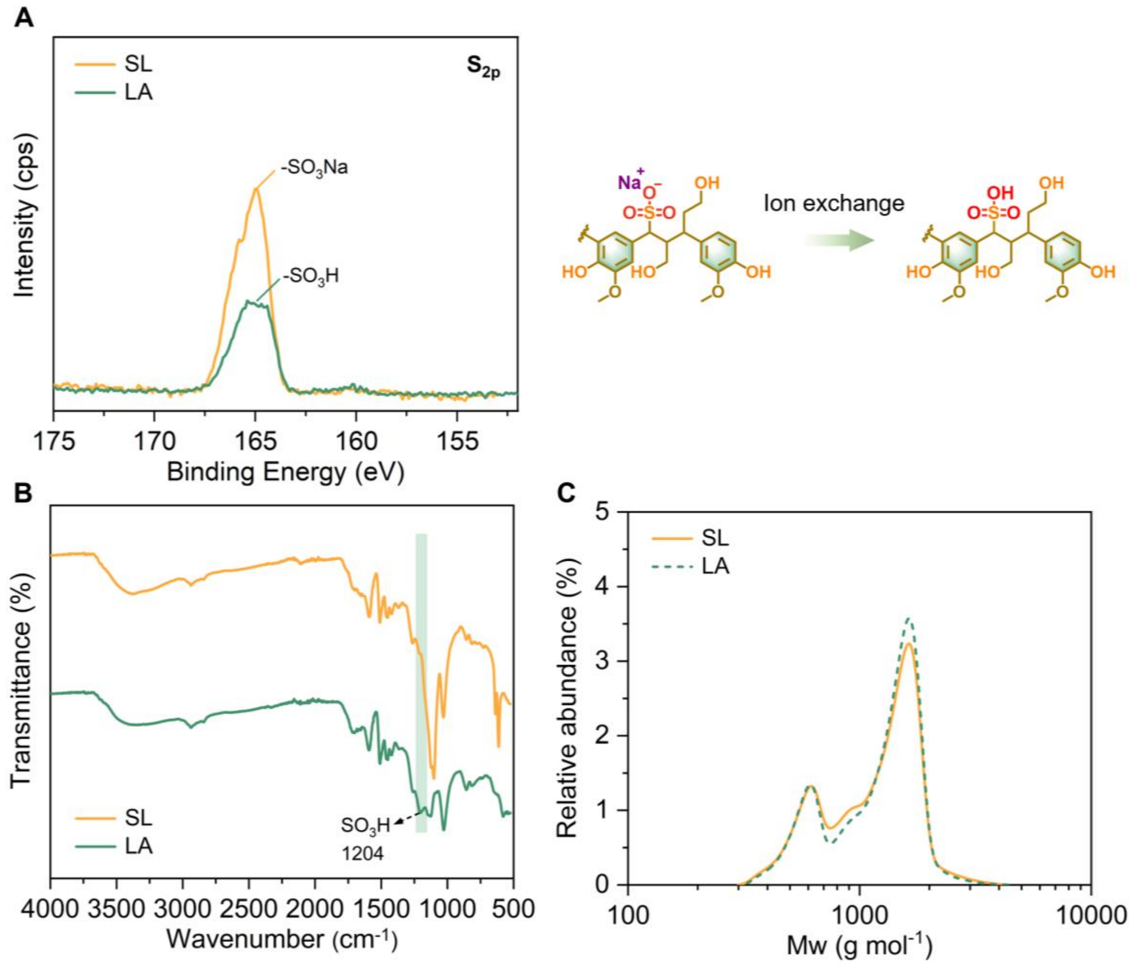

Fig. S5. Characteristics and structure of SL and LA samples. (A) High-resolution S_{2p} spectra, (B) FT-IR absorption spectra and (C) molar mass distribution of SL and LA samples.

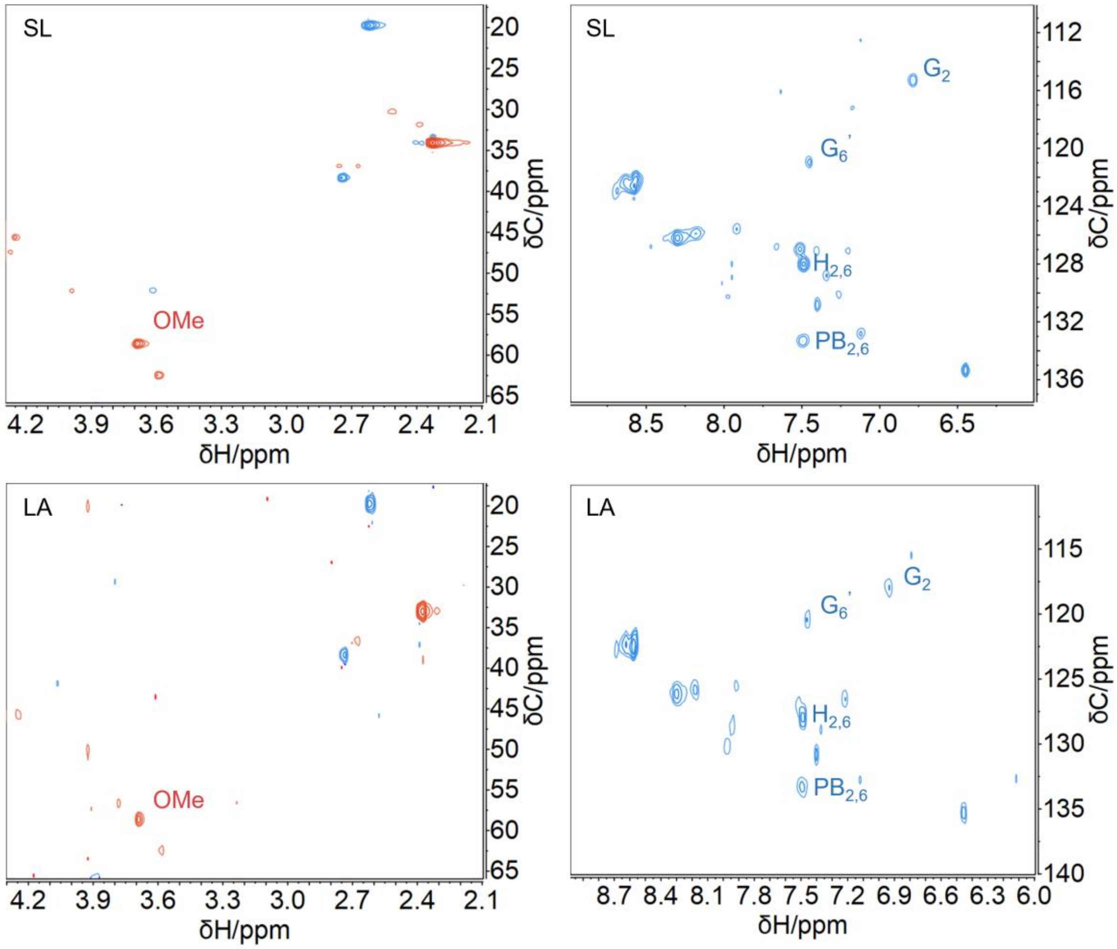

Fig. S6. ^{1}H-^{13}C NMR spectra of the SL and LA samples.

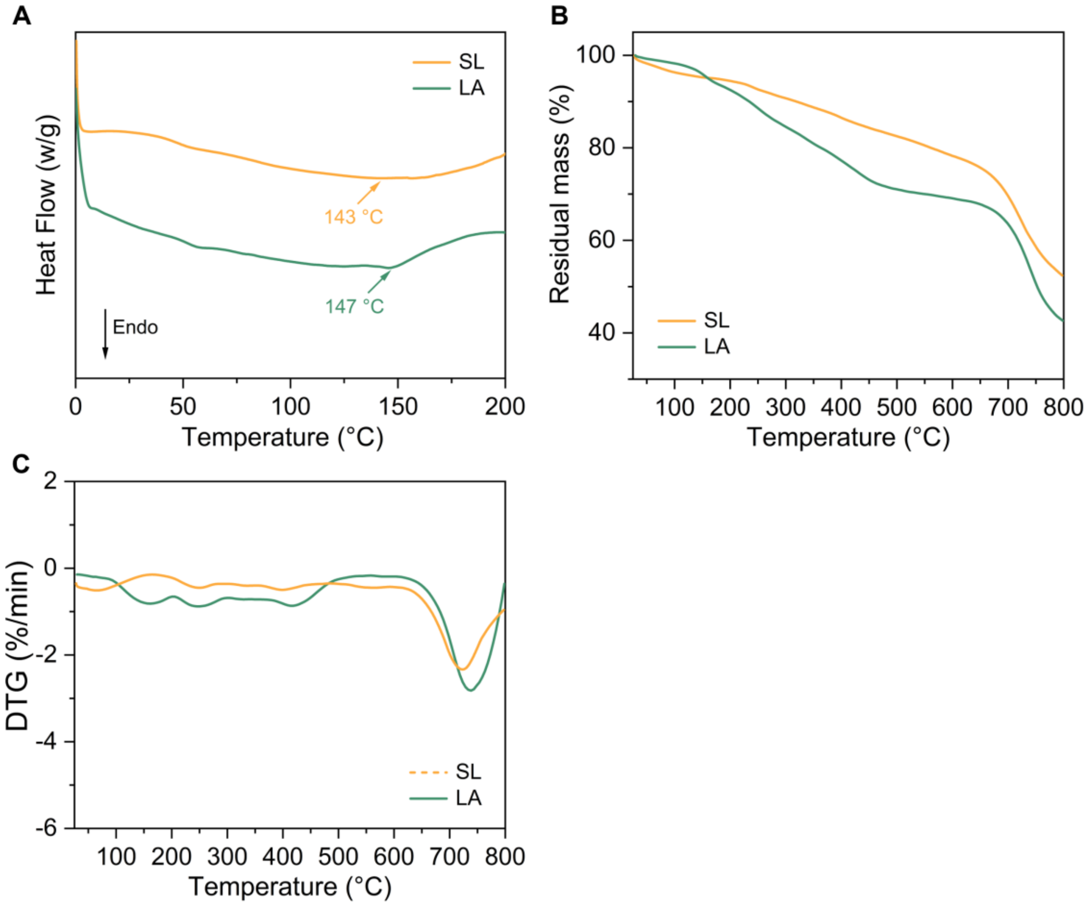

Fig. S7. Thermal stability of SL and LA samples. (A) DSC, (B) TGA, and (C) DTG curves of SL and LA samples.

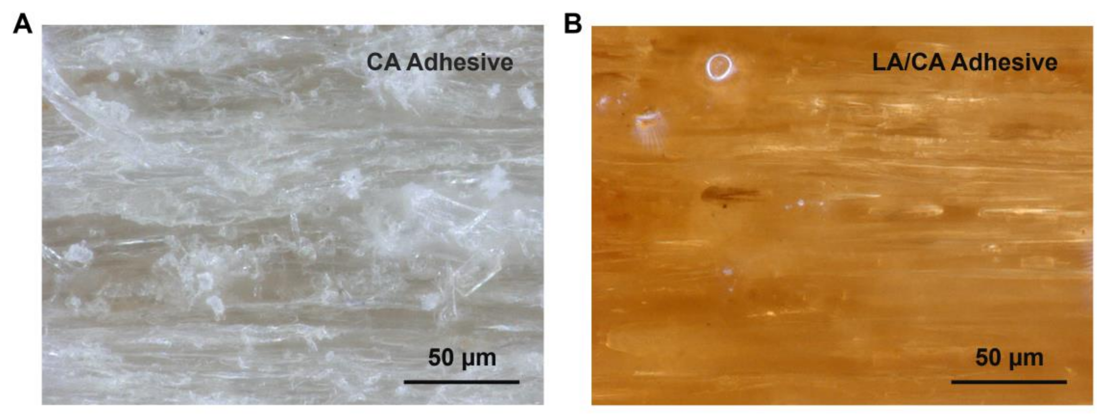

Fig. S8. The super-depth-of-field microscope image of CA (A) and LA/CA (B) adhesives on the surface of wood.

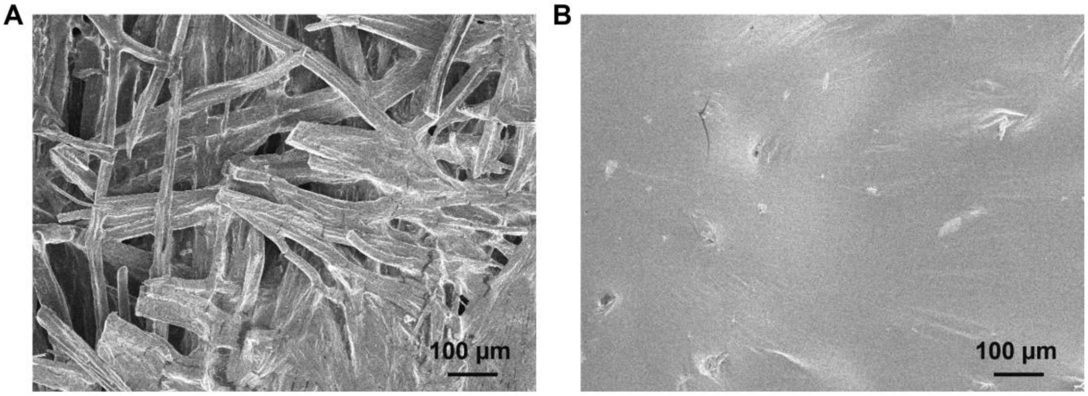

Fig. S9. The SEM image of CA (A) and LA/CA (B) adhesives on the surface of wood.

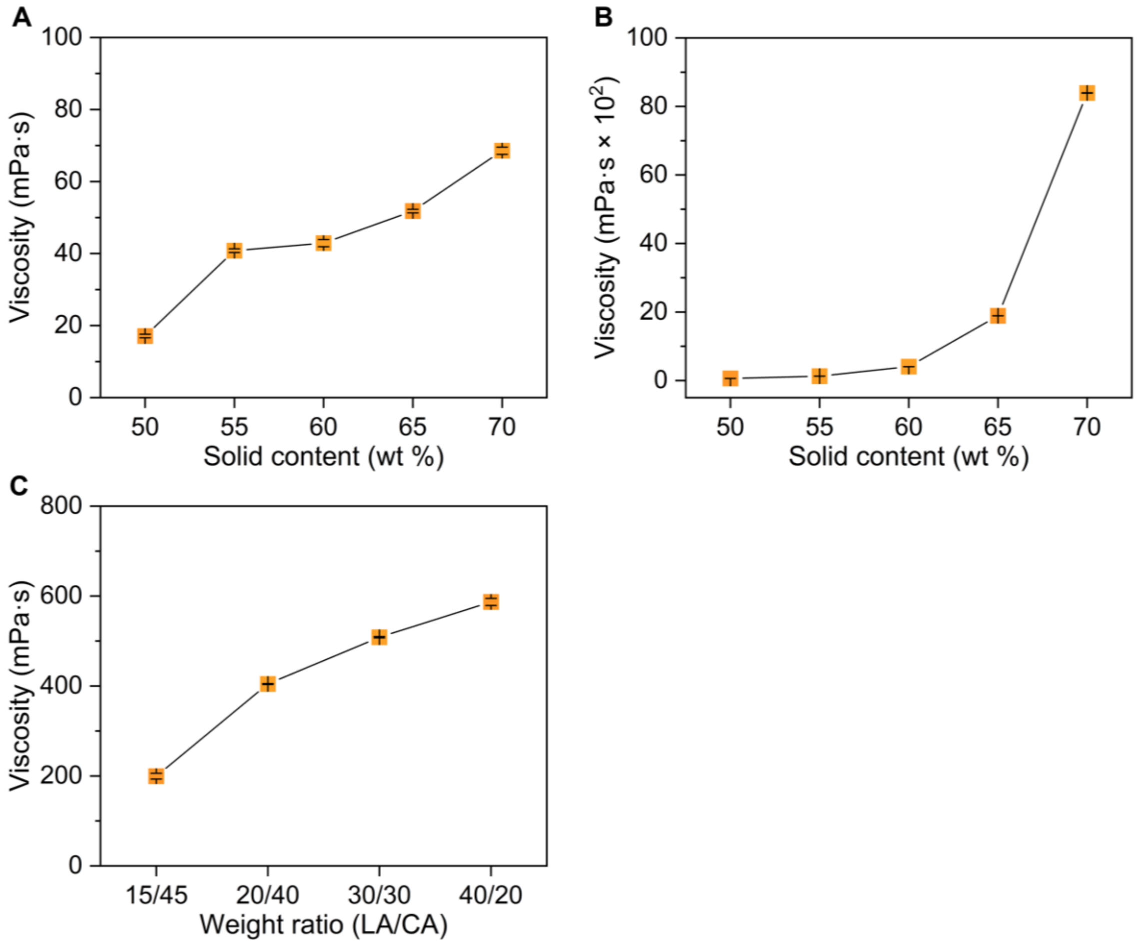

Fig. S10. The viscosity test of CA and LA/CA adhesive. (A) The viscosity test of CA and (B) LA/CA adhesive with different solid content (50-70 wt%). (C) The viscosity test of LA/CA adhesive with different LA/CA ratios.

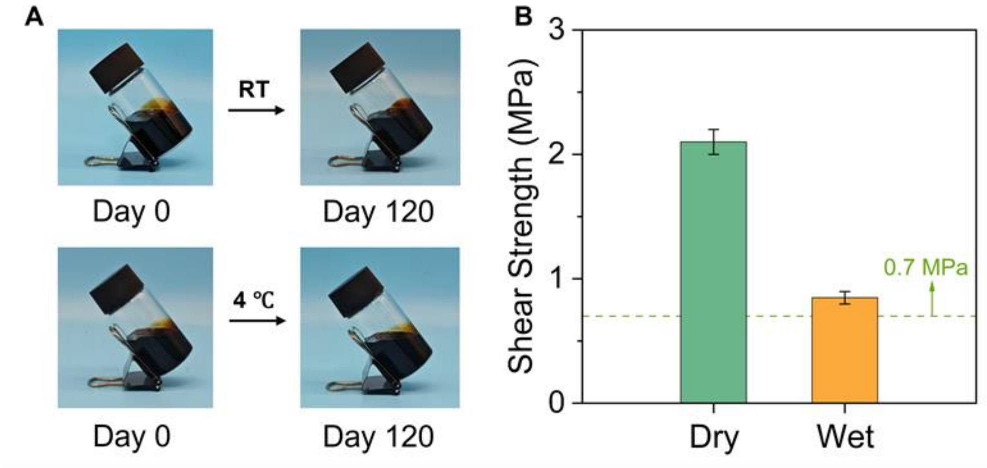

Fig. S11. The storage stability and shelf life of LA/CA. (A) Digital images of the LA/CA adhesive after being stored at room temperature and at 4 ℃ for 120 days. (B) The bonding strength of the LA/CA adhesive stored at room temperature for 120 days.

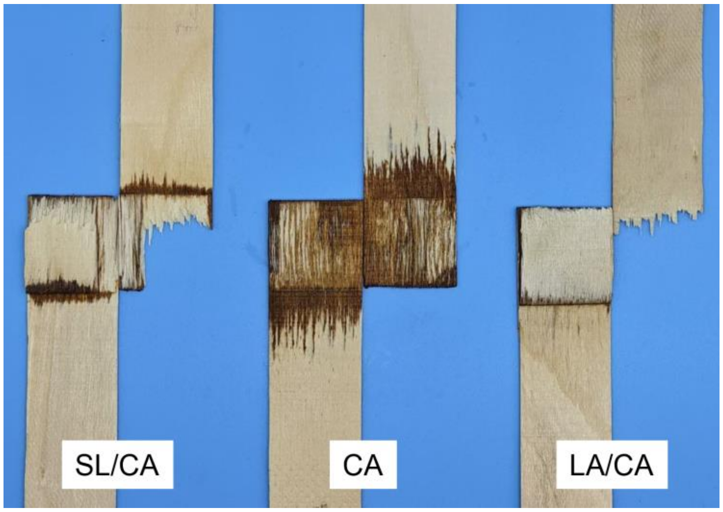

Fig. S12. The digital image showing the failure forms of specimens after the dry shear strength tests of SL/CA, CA and LA/CA.

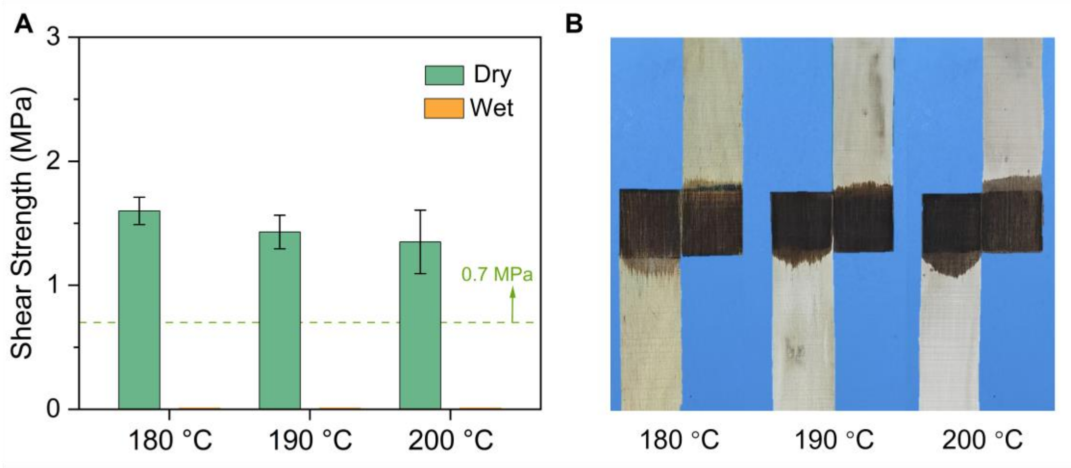

Fig. S13. (A) Dry and wet shear strength of specimen bonded with SL/CA adhesive under different hot-pressing temperatures (180-200 ℃). (B) Images of the failure forms of the specimens prepared under hot pressing temperatures of 180, 190 and 200 ℃ respectively after the wet strength test.

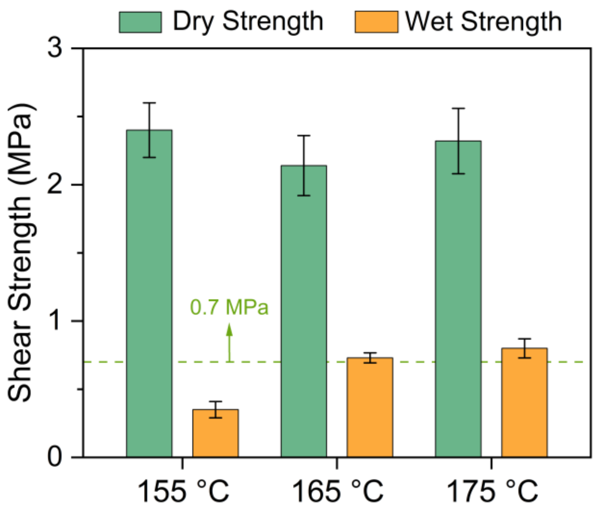

Fig. S14. Dry and wet shear strength of specimen bonded with LA/CA adhesive under different hot-pressing temperatures (155-175 ℃).

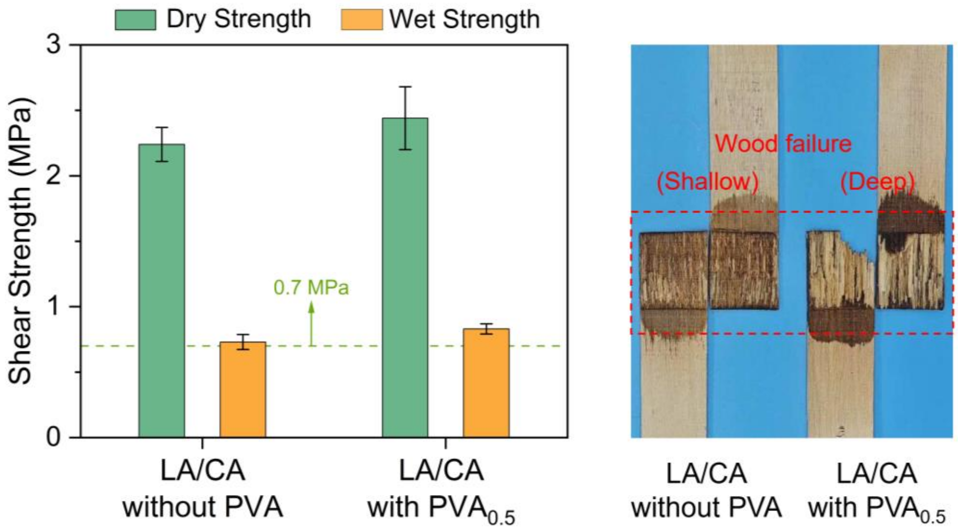

Fig. S15. The shear strengths and failure forms of the bonded specimens prepared with LA/CA adhesive with and without the addition of PVA (0.5 wt%).

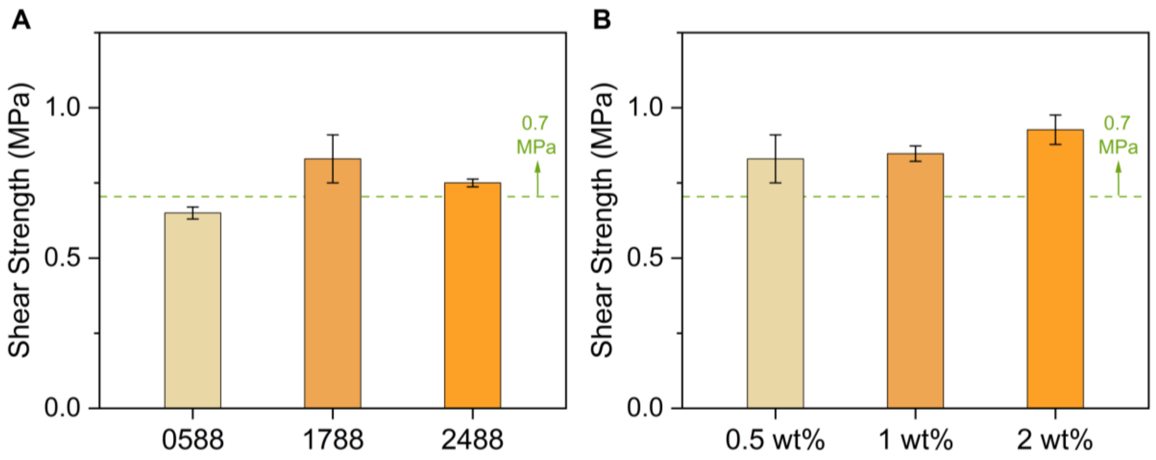

Fig. S16. The wet shear strength of the specimens with different (A) molecular weights (0588, 1788, 2488) and (B) quantities (0.5-2.0 wt%) of PVA added.

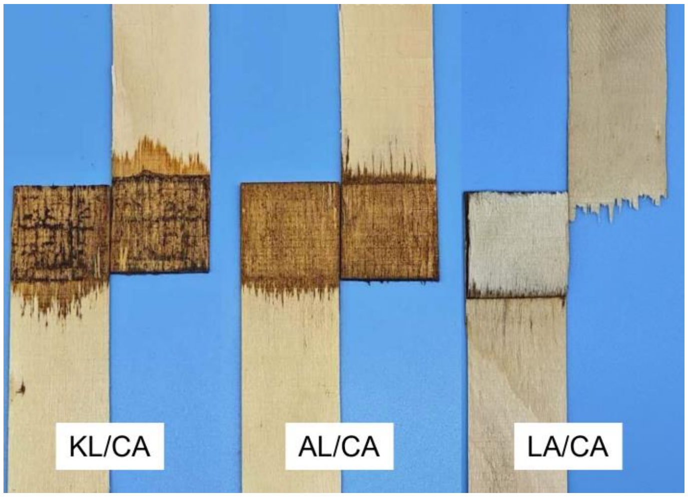

Fig. S17. The digital image showing the failure forms of specimens prepared with KL/CA, AL/CA and LA/CA adhesives.

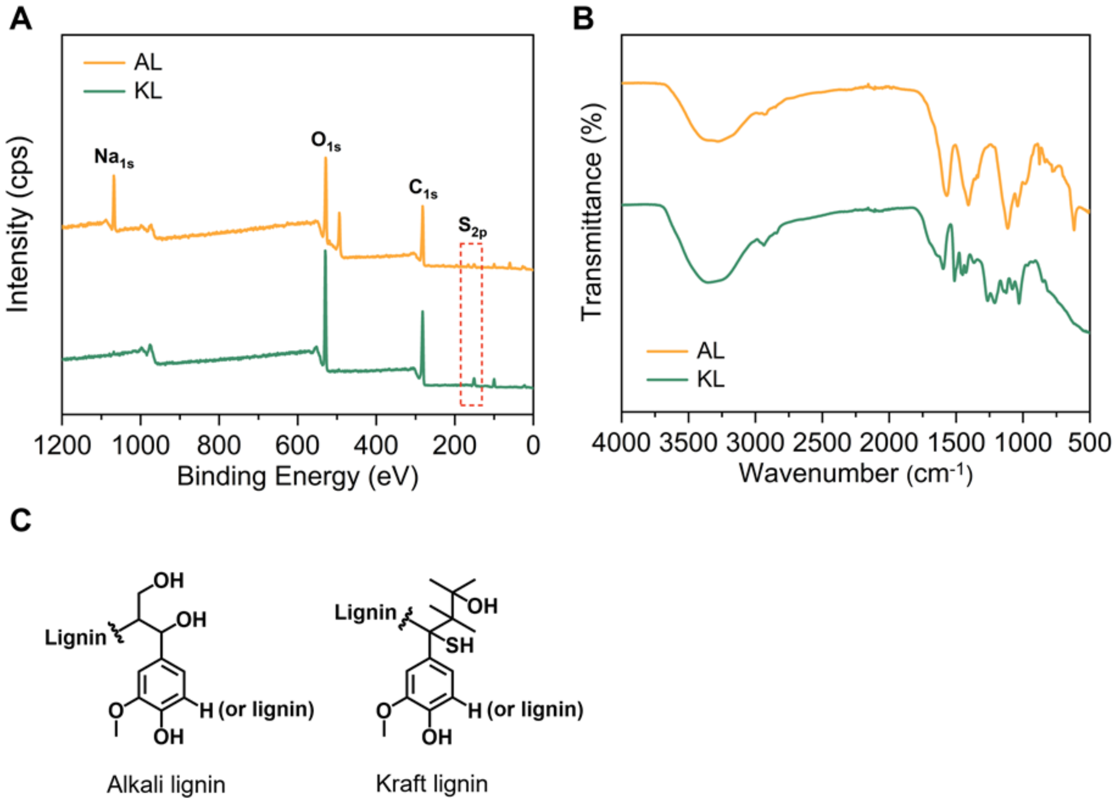

Fig. S18. Structural characterization of AL and KL. (A) XPS spectra and (B) FT-IR absorption spectra and (C) The structural schematic of AL and KL.

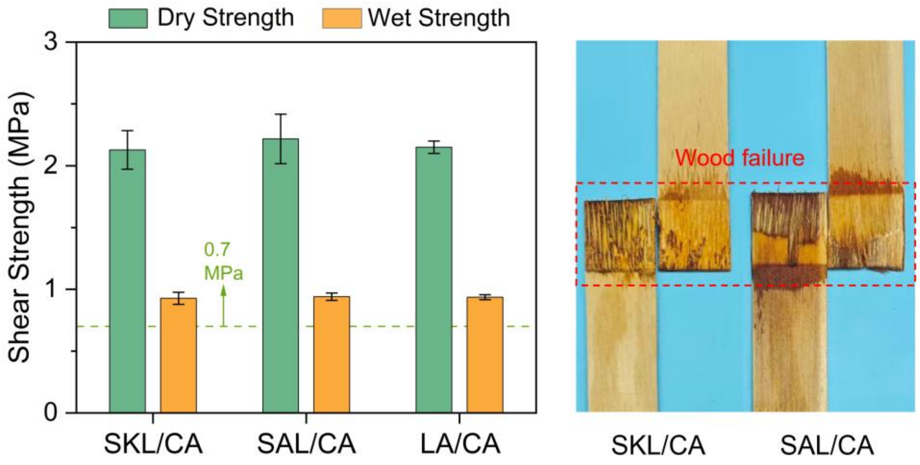

Fig. S19. The shear strengths and failure forms of the bonded specimens prepared with SKL/CA and SAL/CA adhesives.

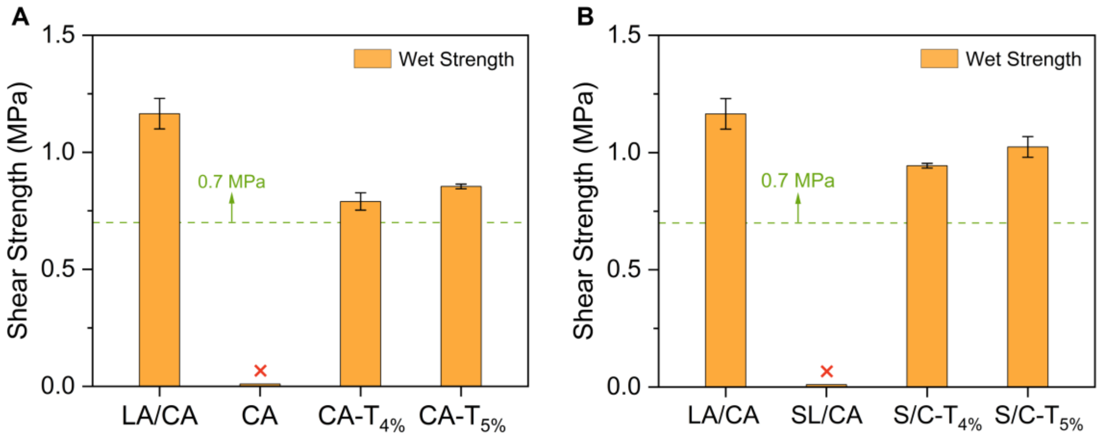

Fig. S20. The wet strengths of the (A) CA (CA-$T_{4\%}$, CA-$T_{5\%}$) and (B) SL/CA (S/C-$T_{4\%}$, S/C-$T_{5\%}$) adhesives with TsOH compared with the LA/CA adhesive.

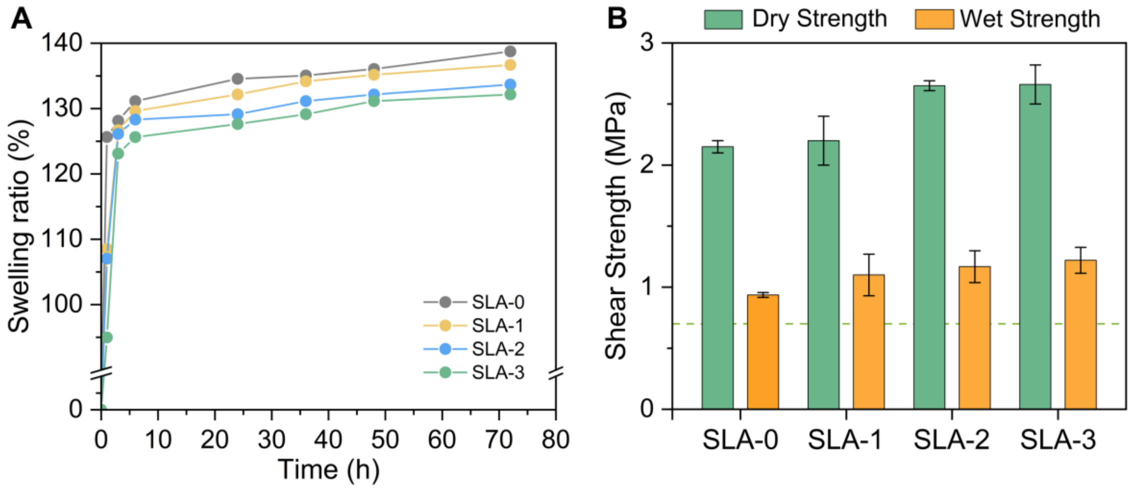

Fig. S21. (A) The swelling ratio and (B) dry-wet shear strength of SLA.

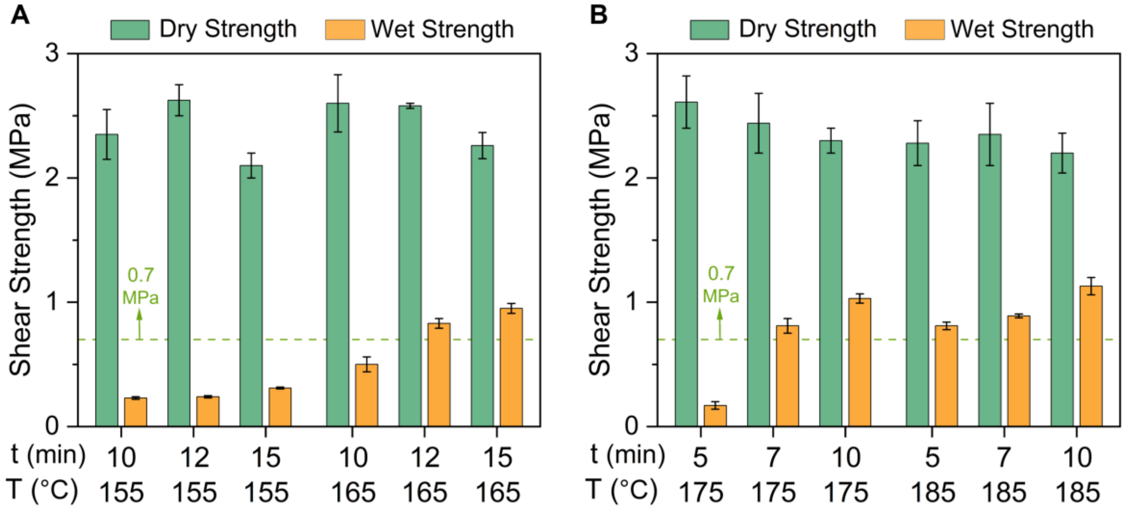

Fig. S22. Dry and wet shear strength of specimen bonded with LA/CA adhesive under different hot-pressing temperatures and times.

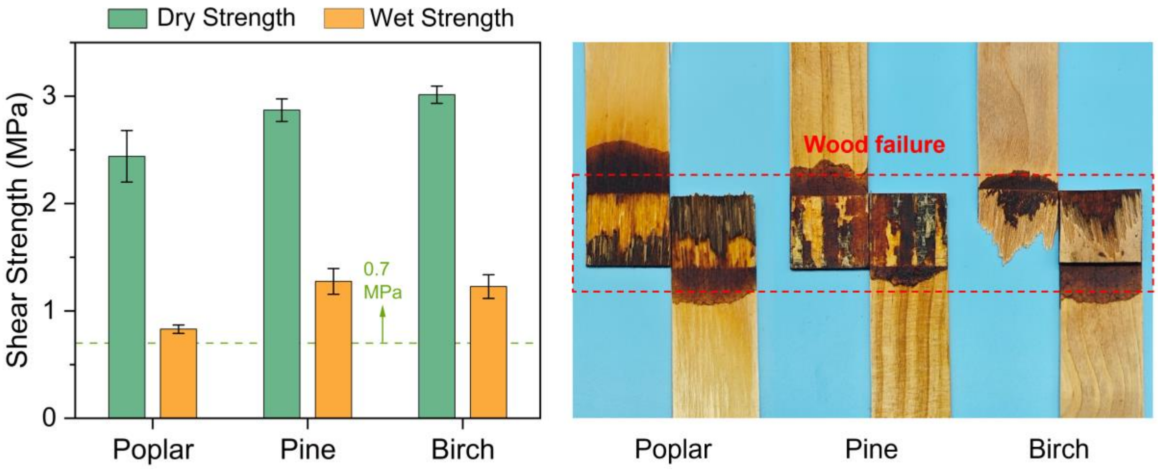

Fig. S23. The shear strength and failure forms of different types of wood specimens bonded by LA/CA adhesive.

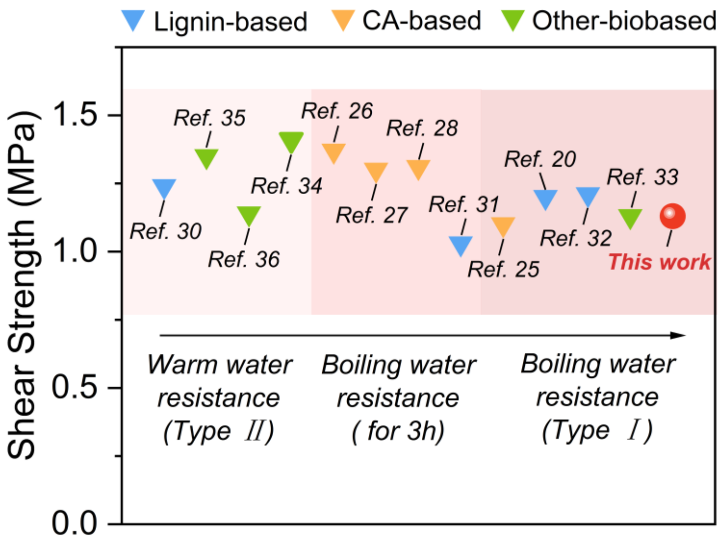

Fig. S24. This study was compared with the performance (water resistance) of previously reported lignin-based, citric acid-based and other biobased adhesives.

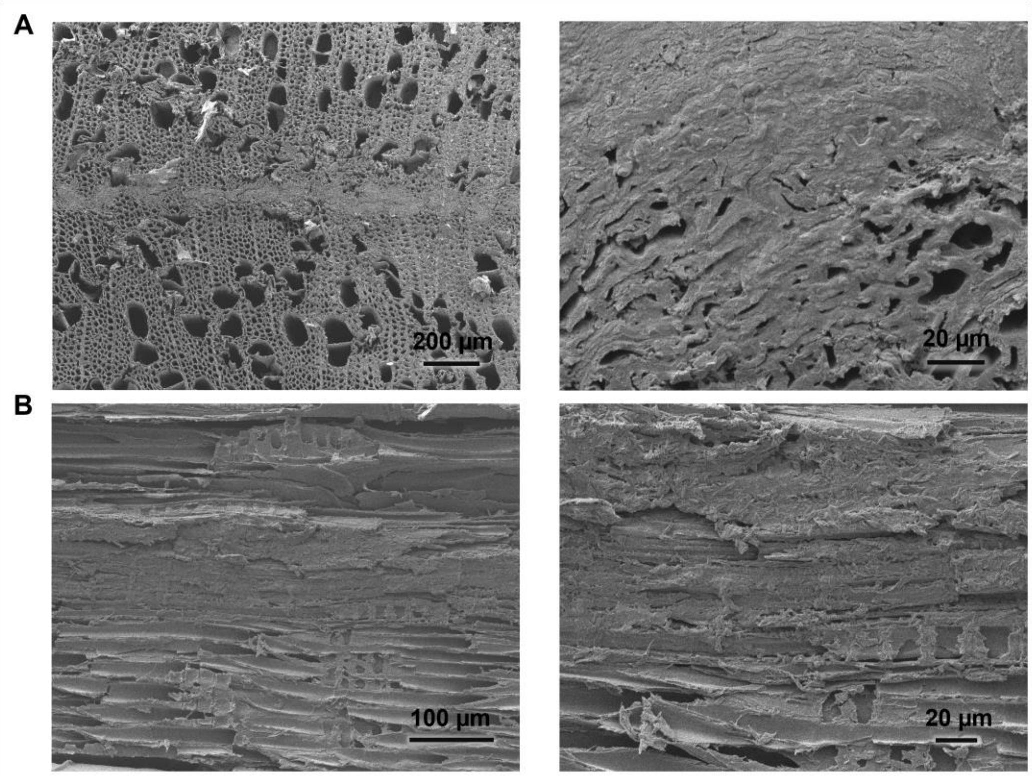

Fig. S25. The SEM images of the (A) cross-sectional and (B) longitudinal sections of the plywood after boiling.

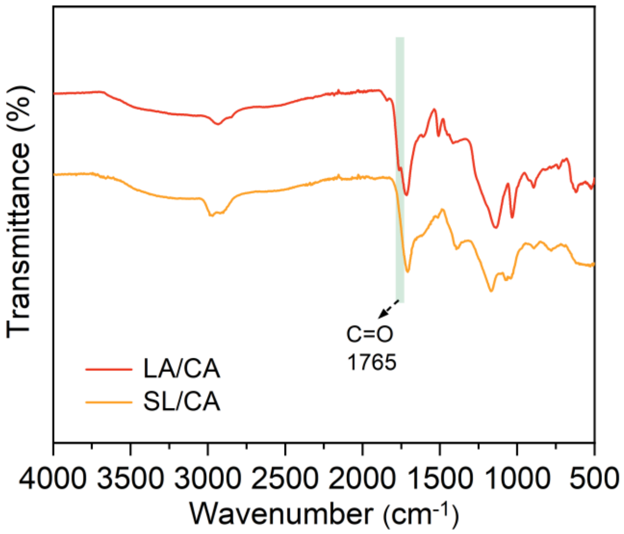

Fig. S26. FT-IR absorption spectra of LA/CA and SL/CA for the same condition.

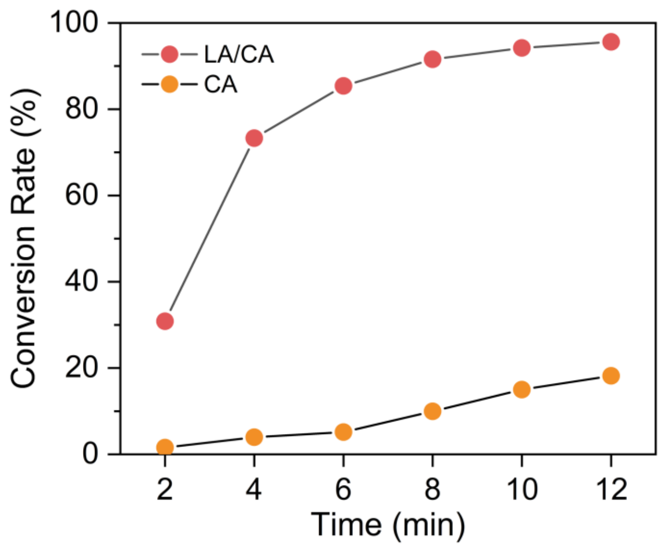

Fig. S27. The conversion rates of CA and LA/CA adhesives at different times.

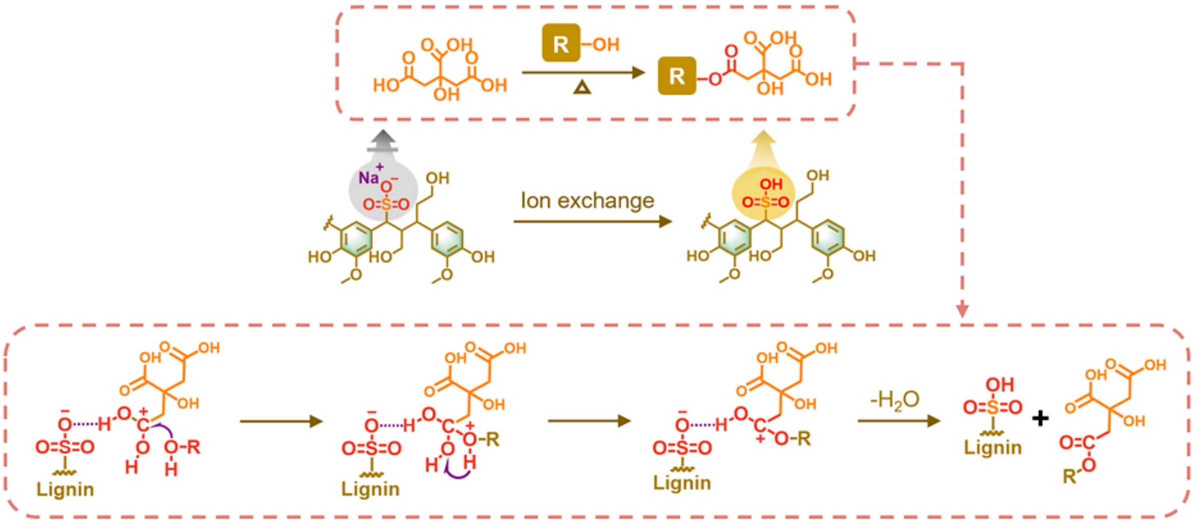

Fig. S28. The catalytic esterification mechanism of lignosulfonic acid.

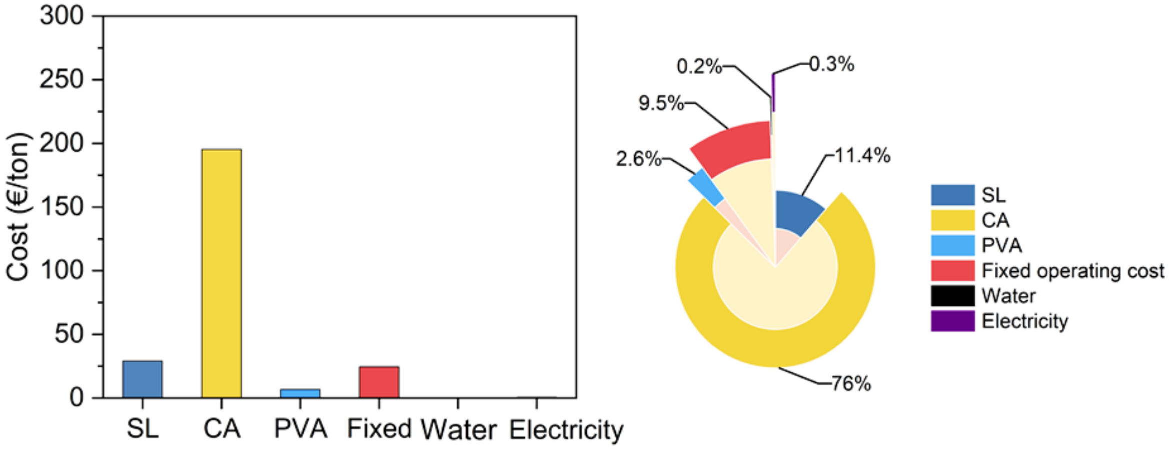

Fig. S29. Costs and Percentage distribution for producing 1 ton of the LA/CA adhesive.

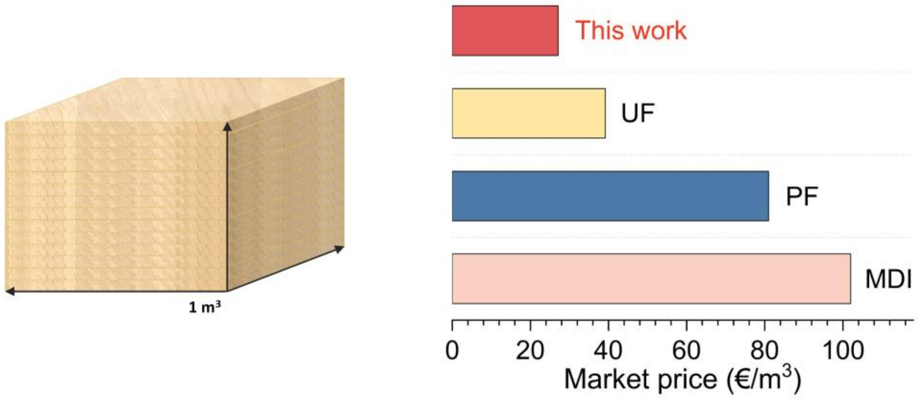

Fig. S30. Comparison of the cost of LA/CA adhesive and traditional adhesive (UF, PF and MDI resins) used for producing 1 m^3 plywood.

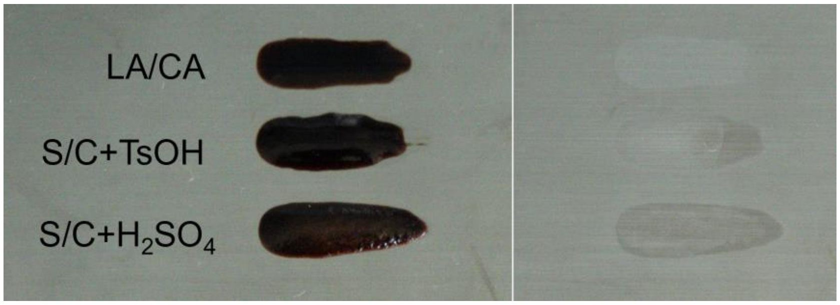

Fig. S31. Corrosion tests for LA/CA adhesive, SL/CA adhesive with TsOH and H_2SO_4.

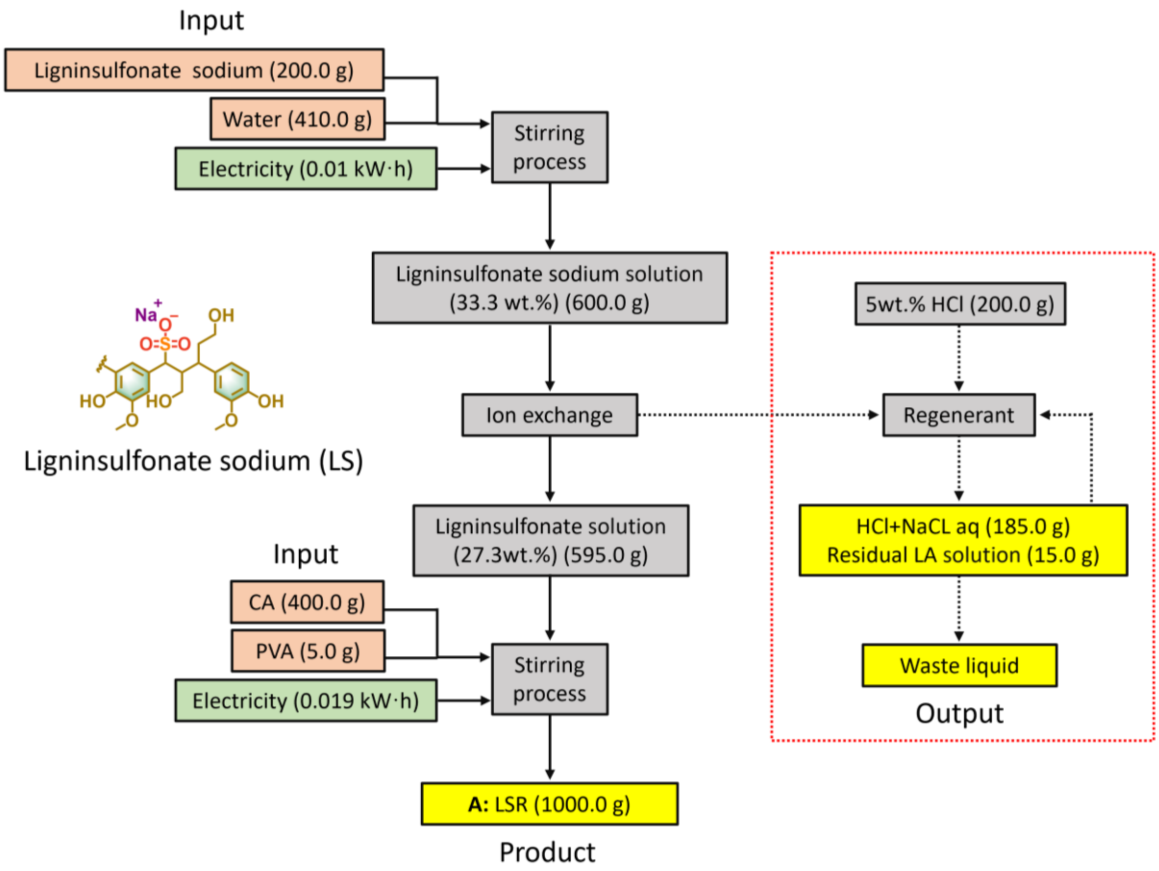

Fig. S32. System boundary diagram of the life cycle assessment.

2. Supplementary Tables

Table S1. Effect of ion exchange treatment on the elemental compositions.

Sample	N (%)	C (%)	H (%)	S (%)
SL	1.15	42.63	4.38	6.95
LA	1.15	42.54	4.39	6.60

Table S2. The viscosity test of CA adhesive with different solid contents.

Sample	Solid content (wt%)	Viscosity (mPa.s)
CA adhesive	50	17
	55	41
	60	43
	65	52
	70	68

Table S3. The viscosity test of LA/CA adhesive with different solid contents.

Sample	Solid content (wt%)	Viscosity (mPa.s)
LA/CA adhesive	50	62
	55	127
	60	404
	65	1882
	70	8393

Table S4. The -SO_3H density and crosslinking density of SLA.

Sample	Sulfonation times (h)	-SO_3H density (mmol/g)	Crosslinking density (mol/cm^3)
SLA-0	0	1.28	14.28×10^{-3}
SLA-1	3	1.40	15.74×10^{-3}
SLA-2	4	1.65	17.93×10^{-3}
SLA-3	5	1.85	18.50×10^{-3}

Table S5. This study was compared with the performance of previously reported lignin-based, citric acid-based and other biobased adhesives.

	Matrix	Dry strength (MPa)	Wet strength (MPa)	FA emission	Sustainability	Ref.
This work	CA/lignin	2.44	1.13/Type I	0.11	high	This work
CA/chitosan	CA/CS	2.10	1.10/Type I	-	high	25
MM-CACS	CA/CS	-	1.37/93 ℃,3h	0	high	26
CAG	CA/glucose	1.50	1.35/93 ℃,3h	0	high	27
CHP	CA/cellulose	2.24	1.19/93 ℃,3h	-	high	28
LPF	Lignin	1.45	1.17/Type I	0.2	middle	20
LPF	Lignin	1.33	1.24/Type II	-	middle	30
OSL	Lignin	1.04	1.03/93 ℃,3h	-	high	31
DLPF	Lignin	2.46	1.21/Type I	-	middle	32
PA-MACS	MA/CS	1.66	1.13/Type I	-	high	33
SPI-10RA-6BCNF-4MMT	SPI	2.41	1.41/Type II	-	high	34
ESM/GOx	soyabean	2.15	1.35/Type II	-	high	35
SMAP	soyabean	2.03	1.14/Type II	-	high	36

Table S6. LA/CA adhesive production operating costs.

Component	Price	Cost	
Sodium ligninsulfonate (SL)	146.55	29.31	€/ton
Citric Acid (CA)	554.37	221.75	€/ton
Polyvinyl alcohol (PVA)	1341.98	6.71	€/ton
Water	0.29	0.12	€/ton
Electricity	0.06	0.60	€/ton
Total		232.17	€/ton
Fixed operating cost		24.43	€/ton
Total		256.60	€/ton

Table S7. Detailed operating costs for producing 1 ton of the LA/CA adhesive.

Raw material	Quantity/Energy	Unit	Price
Step 1: SL solution preparation			
Sodium ligninsulfonate (SL)	200	kg	29.31 €/ton
Water	420	kg	0.12 €/ton
Electricity	1	kWh	0.06 €/ton
Cost: 29.31+0.12+0.06 = 29.49 €/ton			
Step 2: Ion exchange process			
Electricity	7	kWh	0.42 €/ton
Cost: 0.42 €/ton			
Step 3: LA/CA preparation			
Citric Acid (CA)	400	kg	221.75 €/ton
EnergyPolyvinyl alcohol (PVA)	5	kg	6.71 €/ton
Electricity	2	kWh	0.12 €/ton
Cost: 221.75+6.71+0.12 = 228.58 €/ton			
Fixed operating cost			
Ion-exchange resin lifetime	1.5	year	11.2 €/ton
Average regeneration efficiency	92%		
Labor costs			8.13 €/ton
Waste handling	8	€/ton	1.5 €/ton
Cost: 11.2+8.13+1.5 = 20.83 €/ton			
Total Cost: 29.49+0.42+228.58+20.83 = 279.32 €/ton			

Note: The price per ton of water is 0.29 €/ton and the price of electricity is 0.06 €/kWh in Harbin, China.

Table S8. Cost sensitivity analysis of the LA/CA adhesive.

Sensitive variable	Fluctuation range	Cost (€/ton)	Total cost (€/ton)
Baseline	-	-	279.32
SL	+10%	32.24	282.25
	-10%	26.38	276.39
CA	+10%	243.92	301.49
	-10%	199.57	257.14
Ion-exchange resin lifetime	+50% (2 year)	12.32	280.44
	-50% (1 year)	10.08	278.20
Labor costs	+10%	8.94	280.13
	-10%	7.32	278.51

Table S9. Detailed operating costs for producing 1 m^3 plywood with LA/CA, UF, PF and MDI.

Adhesive	Price (€/kg)	Glue-spread (g/m^2)	Glue-spread (kg/m^3)	Cost (€/m^3)
UF	0.24	220	154	36.96
PF	0.52	160	106	55.12
MDI	1.46	110	67	97.82
This work	0.28	160	106	29.68

Table S10. The uncertainty analysis of LA/CA.

Impact category	Unit	Mean	Median	SD	CV	2.50%		97.50%
Fossil resource scarcity	kg oil eq	4.75E-01	4.69E-01	2.60E-02	5.47E-02	4.39E-01	5.40E-01	2.41E-06
Freshwater eutrophication	kg P eq	1.01E-03	9.98E-04	6.50E-05	6.43E-02	9.33E-04	1.16E-03	1.66E-04
Global warming	kg CO_2 eq	3.05E+00	3.04E+00	3.39E-02	1.11E-02	2.99E+00	3.13E+00	2.05E-06
Human carcinogenic toxicity	kg 1,4-DCB	3.45E-01	3.43E-01	1.09E-01	3.17E-01	1.40E-01	5.70E-01	1.07E-03
Marine ecotoxicity	kg 1,4-DCB	1.83E-01	1.82E-01	5.96E-03	3.26E-02	1.75E-01	1.96E-01	2.10E-03
Mineral resource scarcity	kg Cu eq	8.73E-03	8.70E-03	1.47E-04	1.68E-02	8.51E-03	9.09E-03	3.52E-07
Ozone formation, Human health	kg NO_x eq	5.54E-03	5.53E-03	8.39E-05	1.52E-02	5.38E-03	5.71E-03	4.64E-06
Ozone formation, Terrestrial ecosystems	kg NO_x eq	5.67E-03	5.67E-03	8.61E-05	1.52E-02	5.51E-03	5.85E-03	2.65E-06
Terrestrial ecotoxicity	kg 1,4-DCB	2.07E+01	2.04E+01	1.49E+00	7.19E-02	1.89E+01	2.49E+01	3.72E-06
Water consumption	m^3	3.38E-02	5.16E-02	3.96E-01	1.17E+01	-8.47E-01	7.83E-01	4.72E-02

The uncertainty analysis results were obtained through the Simapro Monte Carlo simulation (1000 times) function, including statistical parameters such as the mean value, median value and confidence interval. The results show that there are significant differences in the environmental impacts of LSR production. The global warming potential is 3.05 kg CO_2 eq, with a relatively low uncertainty (CV=1.11%). The uncertainties for water consumption (CV=11.70%) are extremely high, with large differences between the median and the mean value, indicating a highly skewed data distribution. The uncertainties for freshwater eutrophication and marine ecotoxicity are moderate (CV=2–4%), while the uncertainties for fossil resource scarcity and terrestrial ecotoxicity are relatively high (CV=5–7%).

Table S11. LCA inventory for LA/CA production.

Impact category		
Sodium ligninsulfonate	Sodium ligninsulfonate	Self-mode
Water	Water, deionised {RoW}| market for water, deionised | Cut-off, U	Ecoinvent
Electricity	Electricity, high voltage {CN}| market group for electricity, high voltage | Cut-off, U	Ecoinvent
Citric acid	Citric acid {GLO}| market for citric acid | Cut-off, U	Ecoinvent
PVA	Polyvinyl alcohol	Self-mode
Electricity	Electricity, high voltage {CN}| market group for electricity, high voltage | Cut-off, U	Ecoinvent
Wastewater	Wastewater, average {RoW}| market for wastewater, average | Cut-off, U	Ecoinvent
